# Character variation of root space microbial community composition in the response of drought-tolerant spring wheat to drought stress

**DOI:** 10.3389/fmicb.2023.1235708

**Published:** 2023-09-15

**Authors:** Jing Fang, Shuli Wei, Yanrong Gao, Xiangqian Zhang, Yuchen Cheng, Jianguo Wang, Jie Ma, Gongfu Shi, Lanfang Bai, Rui Xie, Xiaoqing Zhao, Yongfeng Ren, Zhanyuan Lu

**Affiliations:** ^1^School of Life Science, Inner Mongolia University, Hohhot, China; ^2^Inner Mongolia Academy of Agricultural and Animal Husbandry Sciences, Hohhot, China; ^3^Key Laboratory of Black Soil Protection and Utilization (Hohhot), Ministry of Agriculture and Rural Affairs, Hohhot, China; ^4^Inner Mongolia Key Laboratory of Degradation Farmland Ecological Restoration and Pollution Control, Hohhot, China; ^5^College of Agronomy, Inner Mongolia Agricultural University, Hohhot, China

**Keywords:** drought stress, spring wheat, root-space microorganism, bacterial diversity, fungal diversity

## Abstract

Drought is the most prevalent environmental stress in crop production, posing a significant danger to food security. Microorganisms in the crop root zone affect crop growth and development, enhance effective nutrient use, and resist adversity hazards. To analyze the changes and functional differences of root space microbial (endosphere-rhizosphere-bulk soil) communities in spring wheat under drought stress. In this study, the root, rhizosphere, and bulk soil of the drought-tolerant group (DTG, three varieties) and drought-sensitive group (DSG, three varieties) were collected. The control (CK, 25–28%), moderate drought (MD, 15–18%), and severe drought (SD, 9–12%) were analyzed by high-throughput sequencing and bioinformatics. The results showed significant differences in the diversity of Bacteria and Fungi in the root space of spring wheat under drought stress (*P* < *0.05*), with the drought-tolerant group exhibiting higher microbial diversity. The microbial community change in spring wheat root space was mainly determined by the niche differentiation of endosphere, rhizosphere, and bulk soil and declined from endosphere to bulk soil due to drought. The antagonism between microbial and root-space species increased, and the community’s complexity and stability deteriorated. Enriching drought-resistant preference groups like *Actinobaciota*, *Variovorax*, *Streptomyces*, and *Conocybe* altered the structure and function of the microbial community in the root space of spring wheat. Spring wheat’s root space Bacteria and Fungi have different strategies to respond to drought.

## 1. Introduction

Microorganisms are abundant in soil and are influenced by elements including vegetation type, nutrients, and climate (Leff et al., 2015; Prober et al., 2015; Zhou et al., 2016). Soil microflora microbial communities are micro-ecosystems that regulate and drive several biological processes in the global macro-ecosystem (Bhattacharjee et al., 2022). Earth Microbiome Project (EMP) (Gilbert et al., 2014; Locey and Lennon, 2016; Thompson et al., 2017) showed that the diversity, structure, and function of microbial community were closely correlated with soil environment (Human Microbiome Project Consortium, 2012; Amir et al., 2017). The interaction between crops, soil microenvironment, and soil microorganism maintains the agroecosystem’s equilibrium (Xu et al., 2018). Crop root-space microorganisms include root endosphere (inside the root), rhizosphere (soil close enough to the root system to be affected by the release of root exudates), and bulk soil (soil areas other than the rhizosphere) communities (Naylor and Coleman-Derr, 2017; Francioli et al., 2021), These microorganisms can affect the crops’ growth and development, enhance the effective utilization of nutrients, and resist adversity (Berendsen et al., 2012; Bulgarelli et al., 2013) to improve soil productivity and crop yield. The crops can selectively “acclimate” certain beneficial Bacteria through root exudates, thus enhancing plant growth and development (Reinhold-Hurek et al., 2015; Zhalnina et al., 2018). Improving crop resilience and mitigating adversity hazards through microorganisms is an important means of sustainable agriculture (Zhou et al., 2017).

Drought is the most prevalent environmental stress in crop production, and climate change and global warming are accelerating the occurrence of severe droughts, causing serious ecological and food security problems (Lesk et al., 2016; Webber et al., 2018; Yang N. et al., 2021). Drought will not only affect crop growth and development of crops and the microbial community structure, but it will also increase soil heterogeneity, limit nutrient transport, and raise soil oxygen concentration, resulting in a substantial fall in soil microbial biomass (Xu et al., 2018). Studies have shown that drought can significantly change the microflora structure of Bacteria and Fungi in soil and promote the microorganisms’ enrichment with higher drought tolerance (Ochoa-Hueso et al., 2018). Specific recombination of rice root microbiota distribution was found after drought stress. The proportion of monoderm bacteria such as *Actinobacteria*, *Chloroflexi*, and aerobic *Firmicutes* is substantially higher in root endosphere communities than in rhizosphere populations (Santos-Medellín et al., 2017, 2021). Li et al. (2022) suggested that, under water stress, the relative abundance of *Gemmatimonas* in rhizosphere soil is positively related to the soil water content, while the relative abundance of drought-tolerant microbiota such as *Azospirillum* and *Bradyrhizobium* remained unchanged with the change of soil water content, which potentially used inert strategy to adapt to different levels of water content to resist drought hazard. Several studies have demonstrated that, under drought, plant root microbiota favors migration to gram-positive populations, displacing most gram-negative lineages. The enrichment occurred mostly in the plants’ root endosphere and could also be observed in the rhizosphere. Moreover, this enrichment has also been shown to be positively correlated with the intensity and duration of drought and to disappear rapidly after rehydration (Edwards et al., 2015; Naylor et al., 2017; Fitzpatrick et al., 2018; Ault, 2020). Therefore, to investigate crop resilience mechanisms, it is crucial to comprehend the precise drought-induced alterations in the microbiota of the root space.

Plants benefit from various soil microorganisms, among which root-space microorganisms are vital in plant health, and certain microbiota may mitigate plant drought stress (Santos-Medellín et al., 2021). Plant growth-promoting rhizobacteria (PGPR) can directly or indirectly boost plant growth under stress, inhibit plant pathogenic Bacteria, and enhance plant tolerance to drought by stimulating nitrogen fixation, increasing nutrient absorption, and improving soil properties (Glick, 1995a,1995b; Babalola, 2010). A four-species bacterial population (*Stenotrophomonas rhizophila*, *Xanthomonas retroflexus*, *Microbacterium oxydans*, and *Paenibacillus amylolyticus*, termed SPMX) significantly increased the survival rate of Arabidopsis plants under drought stress and induced drought resistance in plants, which resulted from the potential overall emergence property of SPMX (Yang Y. et al., 2021). Some Fungi colonized in the plants’ endosphere can improve the hosts’ defense against abiotic and biotic stresses and promote plant growth (Feng et al., 2017; Latz et al., 2018). In the rhizosphere, beneficial Fungi, *Arbuscular mycorrhizal fungi* (AMFs), can form a connection between the host and soil by an external hyphal network, hence enhancing the adaptability of host plants, increasing their nutrient absorption and improving their productivity under drought stress (Juroszek and Tiedemann, 2013; Kwak and Weller, 2013). Wheat (*Triticum aestivum* L.) is one of China’s three main foods, and its planting area and production are second only to corn and rice. Spring wheat is mainly grown in northern China, with a wide distribution range and a long planting history. However, drought has caused a serious yield reduction. Soil water stress affects changes in fungal and bacterial communities in the spring wheat microbiome, but has a smaller effect on plant genotypes, and communities sensitive to water stress differ significantly among different wheat genotypes (Azarbad et al., 2020). Breitkreuz et al. (2021) conducted drought stress treatment on two winter wheat varieties with different soil types. It was found that the composition of the bacterial community in rhizosphere soil was strongly affected by drought and soil types, and drought reduced the diversity of rhizosphere Bacteria, increasing the relative abundance of drought-tolerant Bacteria. Jochum et al. (2019) to improve the seedling growth of wheat under extreme drought conditions, a microbial community that delays the dry time of wheat seedlings was obtained through 6 rounds of artificial selection using HMME, and these microbiomes mediate changes in the rhizosphere environment, thereby improving the adaptive ability of plants to drought stress. Mavrodi et al. (2018), research results showed that indigenous Phz^+^ rhizobacteria that produce the antibiotic phenazine-1-carboxylic acid (PCA) contribute to the natural inhibition of soil-borne pathogens in wheat. The previous studies on wheat drought resistance mainly focused on the rhizosphere or only concentrated on one aspect of soil microorganisms (Bacteria, Fungi). However, the changes in microbiota and their interactions in wheat root-space (endosphere-rhizosphere-bulk soil) are rarely reported. Therefore, understanding the response of root-space microorganisms to drought stress in drought-tolerant spring wheat would aid in elucidating the potential causes of the spring wheat’s drought resistance.

Three drought-tolerant spring wheat varieties (Dingxi 40, Longmai 36, and Longmai 33) and three drought-sensitive spring wheat varieties (Non-gmai 2, Bamai 12, and Bafeng 5) were used in this study. Rainproof sheds and regulated hydration were used to achieve water content control. Using high-throughput sequencing technology and bioinformatics, we primarily explored the changes in spring wheat root-space microorganisms. We analyzed the crucial function of several edaphic conditions in creating root-space microorganisms. The following hypotheses were proposed: (1) From the endosphere to the bulk soil, the effect of drought stress on root-space microbial diversity declines; (2) Drought impairs the diversity and stability of root-space microorganisms; (3) Critical bacterial and fungal communities from different locations of the root-space (endosphere-rhizosphere-bulk soil) exhibit different response toward drought.

## 2. Materials and methods

### 2.1. Field experiment design

The field experiment was initiated in 2022 at Inner Mongolia Autonomous Region Academy of Agriculture and Animal Husbandry Tenihe Soil Management and Ecological Restoration Scientific Observation and Experimental Station (Tenihe Farm), Inner Mongolia Autonomous Region, China (49°55′N, 120°48′E, 650 m above sea level). The region has a moderately temperate semi-arid continental steppe climate with an average annual precipitation of 373–474 mm. The daily average temperature and precipitation changes during the whole growth period of spring wheat in 2022 are shown in Supplementary Figure 1. The soil type of the cultivated land at the experimental station was black calcium soil, and the water holding capacity in the field was 30–35%, which belonged to the representative of the black soil in the western foothills of the Daxing’anling Mountains in the typical Inner Mongolia agro-pastoral staggered belt. Pre-sowing 0–20 cm soil nutrients: available nitrogen 37.26 mg ⋅ kg^–1^, available potassium 195.83 mg ⋅ kg^–1^, available phosphorus 21.25 mg ⋅ kg^–1^, organic matter 57.82 g ⋅ kg^–1^, and pH 6.8.

It was a two-factor experiment combining three water treatments and two groups of spring wheat varieties. Using the plant water gradient division method proposed by Hsiao (1973), the main factor was three kinds of water treatments: In the 0–20 cm soil layer, control (CK) the soil mass water content was 25–28% (accounting for 70–80% of the field water-holding capacity); moderate drought (MD) the soil mass water content was 15–18% (accounting for 40–50% of the field water-holding capacity); severe drought (SD) the soil mass water content was 9–12% (accounting for 25–35% of the field water-holding capacity) (Ji et al., 2021). The secondary factor was three cultivars (DX40, LM36, and LM33) in the drought-tolerant group (DTG) and three cultivars (NM2, BM12, and BF5) in the drought-sensitive Group (DSG), for various details in Supplementary Table 1. There were 18 treatments; the drought treatment group and the control group were conducted in the dry shed, and the soil mass water content was controlled through rain prevention and water replenishment in the dry shed, which was mechanically sown on May 9, 2022, with a seeding volume of 300 kg ⋅ hm^–2^. The experiment adopted a random block design (Supplementary Figure 2). Each treatment had three replicate plots with a size of 3 × 3 m, a total of 54 plots. The spacing between the plots was 0.5 m, and a protection row with a width of 1 m was set. The drought treatment lasted 30 days from the beginning of the spring wheat jointing period (July 4) to the end of the flowering period (August 2). On July 28, the soil mass water content reached the preset range, and after 5 days (August 2), the soil mass water content of SD, MD, and CK treatment was 9.6, 15.4, and 25.9% (Supplementary Table 8), respectively, and sample collection began. Details of soil mass water content monitoring are shown in Supplementary Figure 3.

### 2.2. Sample collection

According to the sample collection methods of Bulgarelli et al. (2012) and Bai et al. (2022), the samples from this experiment were collected. The specific method was as follows: in each plot, we adopted the 5-point sampling method, the topsoil was removed on the soil surface at the sampling point, and the soil drill was used to take soil samples of 0–20 cm soil layer, and repeat thrice for each treatment. After the soil sample was fully and evenly mixed, it was screened with 1 mm sterile stainless-steel mesh and divided into two parts. A portion of it was placed in a sterile centrifuge tube, frozen in liquid nitrogen, and stored at −80°C for bulk soil microbial diversity sequencing. The remaining portion was shade-dried and placed in a plastic bag containing desiccant for soil microbiological and chemical analysis. Five representative spring wheat plants were randomly selected in the plot; the bulk soil was removed from the spring wheat’s root, the root was shaken to remove the loose soil at the root, and then a sterile brush was used to remove rhizosphere soil from the root surface. After the spring wheat rhizosphere soil samples repeated thrice in the same treatment were fully mixed and evenly, they were screened with 0.5 mm sterile stainless-steel mesh to remove obvious plant roots, and animal remains, and other residues. Afterward, the screened rhizosphere soil was placed in a sterile centrifuge tube, frozen in liquid nitrogen, and stored at −80°C for rhizosphere microbial diversity sequencing. The remaining wheat roots were rinsed with sterile water 3–5 times, dried with filter paper, and cut with sterile scissors. The roots were placed in a sterile centrifuge tube, frozen with liquid nitrogen, and stored at −80°C for subsequent detection of endosphere microbial analysis. Endosphere, rhizosphere, bulk soil Bacteria, and Fungi were sequenced using 16S rRNA and ITS amplicon sequencing techniques. A total of 162 samples [18 treatments × 3 sample types (root, rhizosphere, and bulk soil) × 3 replicates = 162] were collected.

### 2.3. Microbial DNA extraction, PCR amplification, and amplicon sequencing

According to E.Z.N.A.^®^ soil DNA Kit (Omega Bio-tek, Norcross, GA, U.S.), total microbial genome DNA was extracted from spring wheat root and soil samples under different drought treatments. The total DNA quality and concentration were determined by 1% agarose gel electrophoresis and NanoDrop2000 (Thermo Scientific), an ultramicro nucleic acid quantifier.

The hypervariable region V4 of the bacterial 16S rRNA gene was amplified with primer pairs 515F (5′-GTGCCAGCMGCCGC GGTAA-3′) and 806R (5′-GGACTACHVGGGTWTCTAAT-3′) primers (Caporaso et al., 2012). PCR amplification reaction system: 4 μL 5 × FastPfu buffer, 2 μL 2.5 mM dNTPs, 0.8 μL Each Primer (5 μM), 0.4 μL FastPfu polymerase, 0.2 μL BSA, 10 ng of template DNA, and ddH_2_O to a final volume of 20 μL. ITS1F (5′-CTTGGTCATTTAGAGGAAGTAA-3′) and ITS2R (5′-GCTGCGTTCTTCATCGATGC-3′) primers were used to amplify the fungus ITS sequence (Adams et al., 2013). PCR amplification reaction system: 2 μL 10× Buffer, 2 μL 2.5 mM dNTPs, 0.8 μL Each Primer (5 μM), 0.2 μL rTaq Polymerase, 0.2 μL BSA, 10 ng of template DNA, and ddH_2_O to a final volume of 20 μL. PCR amplification condition: 1 × (initial denaturation 95°C 3 min); 27 × (denaturing 95°C 30 s; annealing 55°C 30 s; extension 72°C 45 s); 1 × (single extension 72°C for 10 min). The sample was then kept at 4°C (PCR instrument: ABI GeneAmp^®^ 9700). Purification and quantification were performed using the AxyPrep DNA gel extraction kit (AxyPrep Biosciences, Union City, CA, USA) and quantum™Fluorometer (Promega, USA), respectively. Sequencing was performed by Illumina’s Miseq PE300 platform (Majorbio Bio-Pharm Technology Co., Ltd., Shanghai, China).

### 2.4. Microbial data analysis

The raw sequencing data were processed with fastp (version 0.19.6; Chen et al., 2018) and software splicing FLASH (version 1.2.11; Magoč and Salzberg, 2011), screening out low-quality Reads sequences and splicing the remaining two-terminal sequence data into Tags. Using UPARSE software (version 7.1; Stackebrandt and Goebel, 1994; Edgar, 2013) According to 97% similarity, the sequences after quality control splicing were clustered by operational taxonomic unit (OTU), and chimeras were removed. The OTU matrix was normalized using the trimmed mean of M values (TMM) method in the R-package edgeR (Robinson et al., 2010). Using an RDP classifier (version 2.11; Wang et al., 2007) compared the Silva 16 S rRNA (Quast et al., 2013) and UNITE ITS (Rustgi et al., 2017) gene databases to classify OTUs, and the confidence threshold was 70%. Each sample’s community composition was counted at different species classification levels.

### 2.5. Statistical analysis

The Bacteria and Fungi sequencing data were analyzed on the Majorbio Cloud platform.^1^ The Shannon index of Alpha diversity was calculated using Mothur (v1.30.1; Schloss et al., 2009) software.^2^ Kruskal-Wallis rank sum test was used to analyze the difference between Alpha diversity groups, and GraphPad Prism 9 was used for drawing. One-way ANOVA and linear mixed model (LMM) were used to analyze the effects of drought stress on bacterial and fungal α diversity. R (version 3.3.1) PCoA statistical analysis (bray-Curtis) was used to test the similarity of microbial community structure in spring wheat root space under drought stress, and the PERMANOVA non-parametric test was used to determine if the difference between drought treatment and spring wheat root space microbial community groups was significant (Oksanen et al., 2013). Moreover, linear discriminant analysis effect Size (LEfSe) (Segata et al., 2011)^3^ (LDA > 3.2, *P* < *0.05*) was used to determine the microbial groups with significant differences in genus abundance between different groups. The VPA analysis in the vegan package of R (version 3.3.1) was used to quantitatively evaluate the individual and joint interpretation of soil microbial traits and soil chemical traits on the microbial community differences in spring wheat root space under drought stress. Based on spearman correlation |*r*| > 0.5 *p* < *0.01*, the top 30 genera of Bacteria and Fungi were screened for correlation network analysis in spring wheat root space under drought stress (Segata et al., 2011). The network analysis tool (Assenov et al., 2008) in Cytoscape was used to calculate the node connectivity (Degree), transitivity, clustering coefficient, the average shortest path of the network, and other attributes obtain the pertinent information between microorganism groups and samples and draw a correlation network diagram.

The structural equation model (SEM) was used to explore the possible direct and indirect effects of drought treatment, spatial position, and spring wheat variety category on soil factors and bacterial and fungal diversity (Shannon). CK, MD, and SD treatments used 25, 15, and 9 as soil mass water content variables, and endosphere, rhizosphere, and bulk soil used 1, 2 and 3 as spatial position variables. Moreover, DTG and DSG used 1, 2 as spring wheat variety category variables. Alpha diversity of Fungi and Bacteria was measured by the Shannon index. The SEM model was fitted using IBM SPSS Amos 22.0 (USA). Chi-square (*P* > *0.05*), the goodness of fit index (GFI > 0.90), and root mean square error of approximation (RMSEA < 0.001) (Grace and Keeley, 2006; Bai et al., 2022). FAPROTAX (Louca et al., 2016) and FUNGuild (Nguyen et al., 2016) tools were employed to predict the bacterial and fungal sequencing results function.

## 3. Results

### 3.1. Changes in microbial diversity

This study employed 16S rRNA and ITS amplicon sequencing techniques were used to systematically analyze the bacterial and fungal community structure in the endosphere (RE), rhizosphere (RS), and bulk soil (BS) of spring wheat under drought stress to clarify the drought stress effect on the spatial microbial diversity of spring wheat roots. Moreover, it was further determined whether the spatial microbial diversity of spring wheat roots in drought-tolerant group (DTG) and drought-sensitive group (DSG) was different in response to drought stress. All samples contained 23,559,191 bacterial and 22,157,557 fungal high-quality sequences (Supplementary Table 2). The bacterial and fungal sequences were clustered into 24,336 and 8,338 OTUs, with an average length of 253.03 and 245.70 bp, respectively. The dilution curve of each sample tends to be flat as sequencing data increases, and the amount of sequencing data is reasonable (Supplementary Figure 4).

**FIGURE 1:**
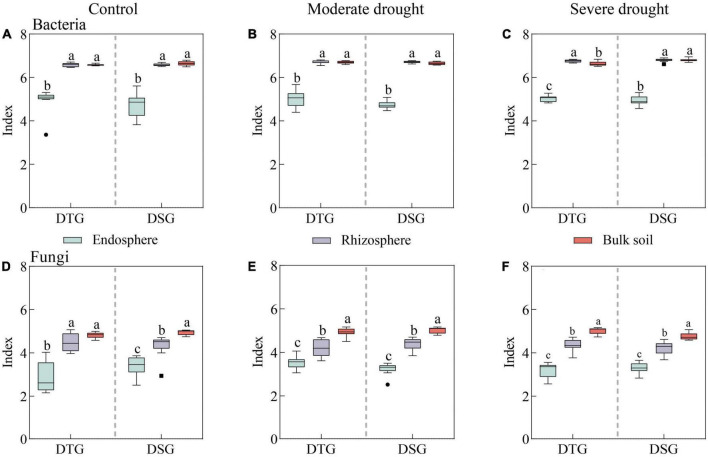
Shannon diversity index (RE, RS, and BS) of Bacteria and Fungi in the rhizosphere of spring wheat under the same drought stress. Shannon index of RE Bacteria **(A)**, Shannon index of RS Bacteria **(B)**, Shannon index of BS Bacteria **(C)**, Shannon index of RE Fungi **(D)**, Shannon index of RS Fungi **(E)**, Shannon index of BS Fungi **(F)**. Endosphere (RE), Rhizosphere (RS), Bulk soil (BS). Drought-tolerant group (DTG) and Drought-sensitive group (DSG).

There were significant differences in the Shannon index of Bacteria and Fungi in root space (RE, RS, and BS) of spring wheat under drought stress (*P* < *0.05*). The bacterial diversity of the rhizosphere and bulk soil was significantly higher than the endosphere under different drought treatments. The fungal diversity was BS > RS > RE, and the Shannon index of endosphere Bacteria and Fungi was the lowest (Figure 1). Moreover, through single factor variance and LMM analysis, it was found that the interaction of Drought treatment × Variety category × Spatial position had a significant effect on fungal community diversity (*P* < *0.01*). There were significant differences in bacterial diversity between drought treatment, rhizosphere, and bulk soil (*P* < *0.01*). The effect of spatial location on bacterial and fungal Alpha diversity was greater than drought treatment (Supplementary Table 3).

In bacterial diversity, compared to CK, there was no significant difference in the Shannon diversity index of spring wheat RE between the drought-tolerant and sensitive group under moderate drought (MD) and severe drought (SD) treatments (*P* > *0.05*) (Supplementary Figure 5A). The Shannon index of RS and BS of spring wheat in the drought-tolerant and the drought-sensitive groups increased slightly with the drought increase (Supplementary Figures 5B, C). Compared to CK, the RE Shannon diversity index of spring wheat in the drought-tolerant group under MD and SD treatments increased initially and then declined (*P* < *0.05*) in terms of fungal diversity (Supplementary Figure 5D). There was no significant difference in RS fungal diversity between the drought-tolerant and the sensitive groups (*P* > *0.05*) (Supplementary Figure 5E). Under SD stress, the drought-sensitive group’s lowest spring wheat BS Shannon index was 4.76 (Supplementary Figure 5F). Drought stress significantly affected the microbial diversity in the root space of spring wheat. Compared to the drought-sensitive group, the drought-tolerant group had higher microbial diversity.

**FIGURE 2:**
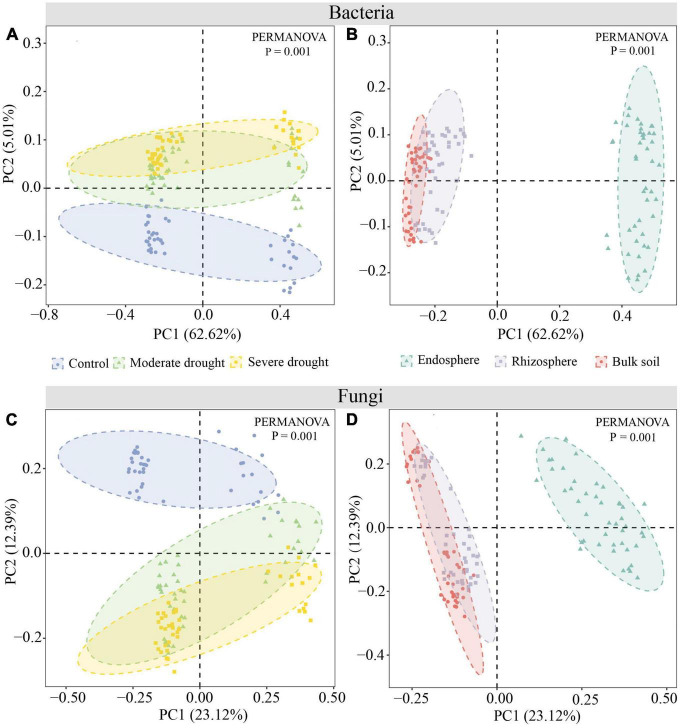
PCoA and Bray-Curtis similarity indexes were used to compare the microbial diversity of spring wheat root space under different drought treatments at the OTU classification level. Bacteria **(A,B)**, Fungi **(C,D)**.

PCoA and PERMANOVA tests revealed significant differences in bacterial and fungal community structure between three water treatments and spatial root positions (*P* < *0.001*). CK samples were significantly distinct from MD and SD samples. Endosphere samples are significantly separated from bulk soil and rhizosphere samples (Figure 2). Furthermore, the PERMANOVA test confirmed that the effect of different drought treatments on fungal (*R*^2^ = 0.17) community composition was greater than Bacteria (*R*^2^ = 0.05). The effects of drought treatment on bulk soil (*R*^2^ = 0.54), rhizosphere (*R*^2^ = 0.45), and endosphere (*R*^2^ = 0.28) fungal communities gradually diminished, although the effect on the rhizosphere (*R*^2^ = 0.39) bacterial community was the greatest (Supplementary Table 4).

### 3.2. Changes in microbial community composition

Analysis of the detected OTUs revealed that drought treatment had significant effects on the endosphere, rhizosphere, and bulk soil community compositions of spring wheat in the drought-tolerant and the drought-sensitive groups (*P* < *0.05*), with the endosphere community composition being more sensitive to drought stress (Supplementary Figures 6, 7). Under drought stress, the relative abundance of *Bacteroidota* and *Proteobacteria* in the bacterial community composition of spring wheat root space gradually increased from bulk soil to endosphere. In contrast, the relative abundance of *Firmicutes* showed the opposite trend. Among the top 10 bacterial phyla, *Patescibacteria* was only enriched in the endosphere. Compared with CK, the relative abundance of *Actinobacteriota* in the endosphere Bacteria of spring wheat in the drought-tolerant group increased by 39.17 and 61.48% under MD and SD treatments, respectively. While the relative abundance in the drought-sensitive group gradually decreased by 20.68 and 12.71%, respectively. Under drought stress, the relative abundance of *Proteobacteria* and *Planctomycetota* in the endosphere and rhizosphere of spring wheat in the drought-sensitive group grew progressively. However, the drought-tolerant group exhibited the reverse pattern (Supplementary Figure 6). At the genus level, the relative abundance of *Variovorax* in the endosphere of spring wheat in the drought-tolerant and drought-sensitive groups increased significantly under drought stress. In contrast, the relative abundance of *Lechevalieria*, *Streptomyces*, *Rhizobacter*, and *TM7a* increased significantly in the drought-tolerant group, and the relative abundance of *Pseudomonas* increased significantly in the drought-sensitive group. The relative abundance of Bacillus in bulk soil and rhizosphere declined steadily with increasing drought, and spring wheat from the drought-sensitive group decreased the most under the SD treatment (RS: 37.42%; BS: 32.01%) (Supplementary Figure 7).

Consistent with the results of bacterial community composition, drought stress also caused significant differences (*P* < *0.05*) in the fungal community composition of spring wheat’s root space (RE, RS, and BS). Under drought stress, *Ascomycota*, *Basidiomycota*, and *Mortierellomycota* were the dominant phyla (Supplementary Figure 6). Compared to CK, the relative abundance of *Basidiomycota*, *Schizothecium*, and *Conocybe* in spring wheat rhizosphere and bulk soil increased gradually under MD and SD treatments. The relative abundance of the pathogenic fungus *Parastagonospora* in the endosphere increased with the drought stress increase, particularly under SD treatment; the sensitive group of spring wheat increased by 986.03% (Supplementary Figure 7). In conclusion, drought stress will alter the microbial community composition in the spring wheat’s root area. *Actinobacteriota* and *Variovorax* are more resistant to drought. The drought-tolerant group strongly prefers *Lechevalieria*, *Streptomyces*, *Rhizobacter*, and *TM7a*, while the drought-sensitive group prefers *Proteobacteria*, *Planctomycetota*, and *Pseudomonas*.

### 3.3. Analysis of differences between microbiomes

**FIGURE 3:**
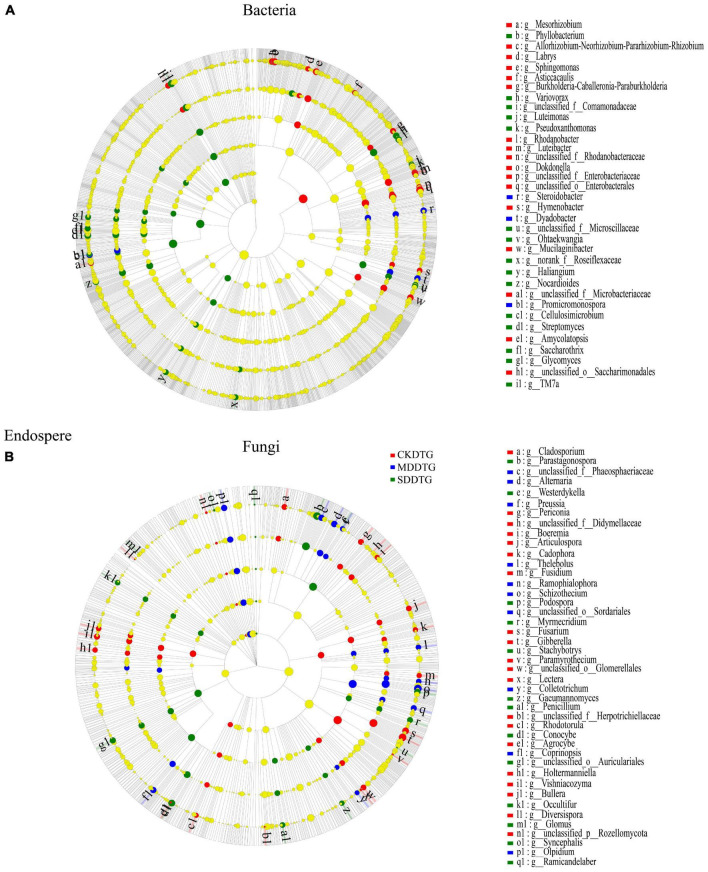
LEfSe analysis of different species between Bacteria **(A)** and Fungi **(B)** in the endosphere of the drought-tolerant group (DRG) spring wheat under drought treatment. Following the species at the phylum, class, order, family, and genus levels is a cladogram from the inner circle to the outer circle. The figure’s letters indicate the species names in the right legend. Different color nodes represent the microbial populations significantly enriched by different treatments, and the differences between groups are significant. The yellow nodes represented the microbial populations with no significant difference between groups (*P* < *0.05*, LDA score 3.2).

Through Lefse analysis, the microbial community differences in root space between the drought-resistant and drought-sensitive groups under drought treatment were explored. At the level of bacterial genus, the biomarkers of endosphere, rhizosphere, and bulk soil of spring wheat in the drought-tolerant and drought-sensitive groups decreased gradually, namely RE-RS-BS: 35-25-14 (Figure 3A; Supplementary Figures 8A, C) and RE-RS-BS: 39-25-9 (Supplementary Figures 9A, C, E), respectively. These biomarkers differed significantly in response to drought treatment (*P* < *0.05*). *Bacillus* was a prevalent biomarker for CK. *Variovorax*, *Streptomyces*, and *Nocardioides* are common biomarkers in the endosphere and rhizosphere of MD and SD spring wheat. *Flavobacterium* and *Pedobacter* were common biomarkers in the rhizosphere of MD spring wheat. In bulk soil, *Blastococcus* under MD treatment and *Massilia* and *Thermomonas* under SD treatment were unique biomarkers for the drought-tolerance group. *Norank* _ *f* _ *Vicinamibacteraceae* and *Norank* _ *f* _ *Nitrososphaeraceae* are unique biomarkers for the drought-sensitive group under MD and SD treatment, respectively. At the fungal genus level, the two groups (DTG and DSG) of spring wheat bulk soil had the most biomarkers, 51 (Figure 3B; Supplementary Figures 8B, D) and 55 (Supplementary Figures 9B, D, F), respectively. *Schizothecium* is a frequent root space fungus biomarker in spring wheat. Consequently, the biomarkers of Bacteria and Fungi in the root space of spring wheat responded differentially to various drought conditions. Under drought stress, *Variovorax*, *Streptomyces*, and other genera in the endosphere and rhizosphere are key biomarkers.

### 3.4. Relationship between microbial community and environmental factors

Spearman correlation analysis was used to analyze the correlation between the endosphere, rhizosphere, bulk soil microbial community, and soil chemical and microbial properties of spring wheat under drought stress in the drought-tolerant group (DTG) and drought-sensitive group (DSG). The results showed that there were six phyla in the spring wheat’s root space in the drought-tolerant group (DTG) that were significantly positively correlated with soil indicators (*P* < *0.05*). They were *Actinobacteriota*, *Proteobacteria*, *Acidobacteriota*, *Chloroflexi*, *Bacteroidota* and *Cyanobacteria*. The relative abundance of *Actinobacteriota* in the endosphere was significantly positively correlated with pH, TK, CAT, and URE. The relative abundance of *Proteobacteria* was positively correlated with MBC, SC, SOC, MBN, and MBP. Significantly good correlations were found between the relative abundance of *Cyanobacteria* in the endosphere of spring wheat in the two groups (DTG and DSG) and ALP (Supplementary Figure 10A). *Basidiomycota*, *Olpidiomycota*, *Mortierellomycota*, *Chytridiomycota*, and *Ascomycota* were significantly positively correlated with soil indicators (*P* < *0.05*) at the phylum level. The relative abundance of *Basidiomycota* in the rhizosphere and bulk soil was significantly positively correlated with TK except in the endosphere of DSG spring wheat (Supplementary Figures 10B, C).

**FIGURE 4:**
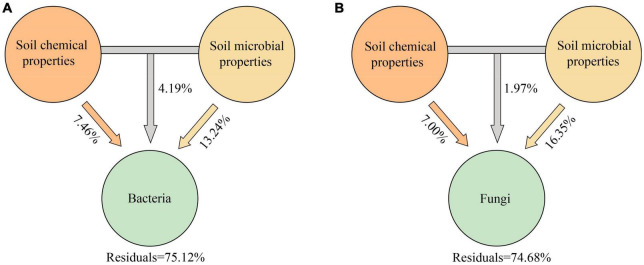
Variance partitioning analysis (VPA) of the effects of soil chemical and microbial properties on rhizosphere Bacteria **(A)** and Fungi **(B)** communities. Soil chemical properties (SCP) include pH; SOC, soil organic carbon content; TP, total phosphorus content; TK, total potassium content. Soil microbiological properties (SMP) include CAT, soil catalase activity; SC, soil invertase activity; URE, soil urease activity; ALP, soil alkaline phosphatase activity; MBC, soil microbial biomass carbon; MBN, soil microbial biomass nitrogen; MBP, soil microbial biomass phosphorus.

**FIGURE 5:**
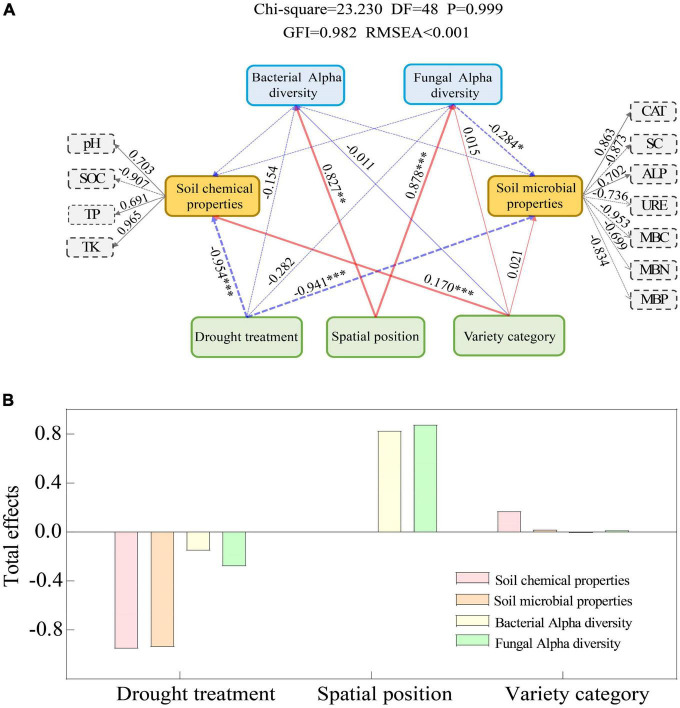
Structural equation model (SME) for direct and indirect effects of drought treatment, spatial position, and spring wheat variety category on soil factors and bacterial and fungal diversity (Shannon) **(A)**. Standardized total effect **(B)**. GIF > 0.9, RMSEA < 0.001 indicated that the model fit well. The red arrow indicates a significant positive impact, the blue arrow indicates a significant negative impact, the thickness of the arrow line indicates the size of the path coefficient in the model, and the dotted line indicates no significant impact; * means *P* < 0.05, ^**^ means *P* < *0.01*, ^***^ means *P* < 0.001.

VPA analysis showed that soil microbiological properties (SMP) were important driving factors for the changes of Bacteria and Fungi communities in the rhizosphere and bulk soil of spring wheat under drought stress (Figure 4; Supplementary Table 5). Soil microbiological properties (SMP) explained 13.24% (Figure 4A) and 14.60% (Supplementary Table 5) of the differences in bacterial community composition between rhizosphere and bulk soil, respectively, while they explained 16.35% (Figure 4B) and 9.97% (Supplementary Table 5) of the differences in fungal community composition, respectively. However, the soil chemical characteristics can only partially explain the discrepancies between the rhizosphere and bulk soil microbial communities (Supplementary Table 5). Afterward, we used the structural equation model (SEM) to analyze the possible pathways of direct and indirect effects of drought treatment, spatial position, and spring wheat variety category on soil factors and bacterial and fungal diversity (Shannon) (Figure 5; Supplementary Table 6). Drought stress had the greatest negative effect on soil chemical properties (−0.945) and soil microbiological properties (−0.941). The spatial position (RE-RS-BS) had the greatest positive effect on the Alpha diversity of Bacteria (0.827) and Fungi (0.878). The spring wheat variety category had the greatest positive effect on soil chemical properties (0.170) (Figure 5). Drought stress harmed bacterial (−0.154) and fungal (−0.282) Alpha diversity. It was found that drought stress had a considerable impact on the Alpha diversity of microorganisms in the root space of spring wheat and that this effect diminished from the endosphere to the bulk soil (Figure 5).

### 3.5. Species correlation network analysis

**FIGURE 6:**
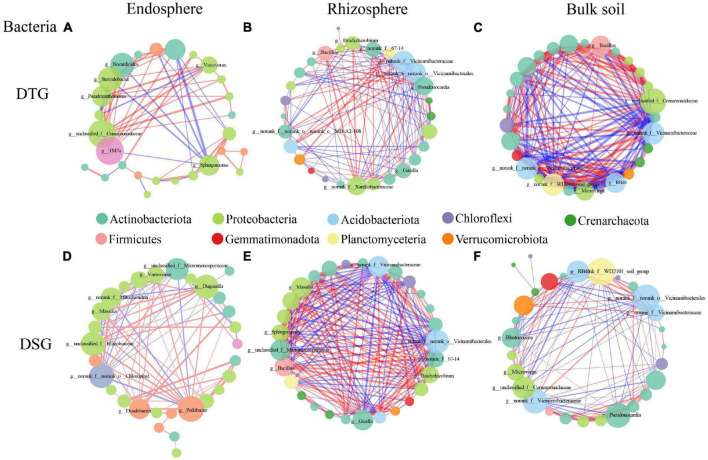
The bacterial genus level correlation network diagram of two groups (DTG and DSG) of spring wheat under drought stress. The red connection indicates a positive correlation, the blue connection indicates a negative correlation, the connection’s thickness represents the correlation coefficient’s size, and the number of lines indicates the degree of connection between nodes.

To further clarify the effect of drought stress on the microbial community in the spring wheat’s root space, we selected the top 30 species at the genus classification level for microbial network construction (Spearman correlation, *P* < *0.01*). The results showed that the network density of bacterial and fungal communities in the root space of the drought-tolerant group of spring wheat under drought stress was BS > RS > RE (Figure 6; Supplementary Figure 11). The maximum network density of the Bacteria rhizosphere in the drought-sensitive group was 0.483, and Fungi results were consistent with the drought-tolerant group (Supplementary Table 7). The positive correlation ratio between rhizosphere and bulk soil bacterial communities in spring wheat 2 groups (DTG and DSG) reduced compared to the endosphere (Figure 6).

In Bacteria, *TM7a* (10) and *Variovorax* (7) in the DTG endosphere; *Pseudonocardia* (15) and *Bacillus* (12) in the rhizosphere; *Bacillus* (18) and *Microvirga* (17) in the bulk soil (Figures 6A–C); in the DSG, *Pedobacter* (13) in the endosphere; *Massilia* (20) and *Bacillus* (19) in the rhizosphere; *Blastococcus* (12) and *Microvirga* (11) in the bulk soil (Figures 6D–F), had the highest degree and positive correlation are both high in the whole network. In Fungi, in the DTG, *Schizothecium* (endosphere, 10), *Occultifur* (rhizosphere, 11), *Coniochaeta* (bulk soil, 18) (Supplementary Figures 11A–C); In DSG, *Parastagonospora* (endosphere, 9), *Subulicystidium* (rhizosphere, 15), *Coniophora* (bulk soil, 19) had the highest degree and the highest sensitivity in the whole network. Most of them were pathogenic Fungi (Supplementary Figures 11D–F). Therefore, *Proteobacteria*, *Actinobacteriota*, *Firmicutes*, *Patescibacteria*, and *Bacteroidota* were the essential core Bacteria of drought stress in the root-space microorganisms of spring wheat 2 groups (DTG and DSG).

### 3.6. Microbial community function prediction

**FIGURE 7:**
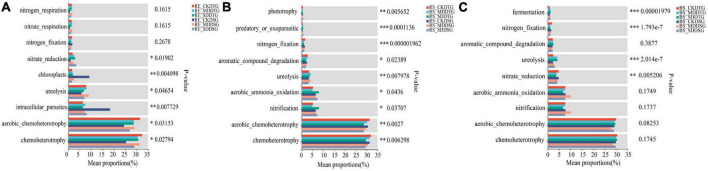
FAPROTAX was used to predict the function of the bacterial community in the root space of spring wheat under drought stress. Endosphere bacterial function prediction **(A)**; Rhizosphere bacterial function prediction **(B)**; Bulk soil bacterial function prediction **(C)**. *P < 0.05, ^**^P < 0.01, and ^***^P < 0.001.

FAPROTAX and FUNGuild tools were used to predict the bacterial and fungal communities’ function in spring wheat root space under drought stress and to explore the functional differences of microbial communities in spring wheat root space under drought stress. The results exhibited that nitrogen fixation was significantly reduced in DTG and DSG (RE-RS-BS) under drought stress in Bacteria (Figure 7). The abundance of nitrate reduction in DTG and DSG spring wheat increased significantly in the endosphere (Figure 7A). Compared to CK, SD dramatically boosted the nitrification abundance of DTG spring wheat by 52% in the rhizosphere (Figure 7B). In bulk soil, the ureolysis abundance of DTG and DSG increased significantly, and DTG increased the most (Figure 7C).

Compared to CK, DTG, and DSG, spring wheat symbiotroph (Arbuscular Mycorrhiza) under MD and SD treatment considerably increased in (RS-BS) (Supplementary Figures 12C–F). In the endosphere, DSG Arbuscular Mycorrhiza of spring wheat decreased significantly, and in the rhizosphere, DSG increased by 1,036–1,463% (Supplementary Figures 12B, D). Fungal Parasite-Plant Pathogen-Plant Saprotroph increased significantly in (RE-RS-BS) (Supplementary Figures 12A, C, E). It suggested that Bacteria and Fungi in the root space of spring wheat responded to dryness using various tactics under drought stress.

## 4. Discussion

### 4.1. Drought-tolerant spring wheat can maintain relatively high microbial diversity

This study explored the microbial diversity of spring wheat roots under drought stress in two groups (DTG and DSG). The bacterial diversity of the rhizosphere and bulk soil was found to be significantly higher than the endosphere under different drought treatments. The fungal diversity was BS > RS > RE, and the Shannon index of endosphere Bacteria and Fungi was the lowest. The results of this study were also confirmed in the study of Xu et al. (2018). Drought stress would significantly reduce the bacterial diversity of the crop endosphere, while the effect on the bulk soil was insignificant. The effect of drought on fungal diversity was consistent with the bacterial diversity results, showing a decreasing trend from the endosphere, rhizosphere, and bulk soil. These results also indicate that the endosphere microbial community of spring wheat is more sensitive to drought stress and more susceptible to drought. Moreover, the Beta diversity PERMANOVA test showed that different drought treatments affected fungal (*R*^2^ = 0.17) community composition more than Bacteria (*R*^2^ = 0.05). Dassen et al. (2017) found that fungal communities are more sensitive to vegetation changes than bacterial communities, and Fungi are also the main consumers of underground plant-derived carbon (Ballhausen and de Boer, 2016). Our results also showed that Fungi are more sensitive to drought than bacteria. It is hypothesized that drought may alter the physiological metabolites of spring wheat and the carbon level of the soil, making fungus more vulnerable to drought. However, the precise reaction mechanism requires additional study.

Our study also found that the drought-tolerant group had higher microbial diversity than the drought-sensitive group under drought stress. Plant species or genotypes can choose different soil communities (Berendsen et al., 2012). Root exudates are vital in the selection of rhizosphere microbial communities (Williams and de Vries, 2020), and rhizosphere microorganisms prefer root exudates (Iannucci et al., 2013). When plants are harmed by adversity, a beneficial rhizosphere microbial community can be selected by modifying the type and amount of root exudates to help the plants survive adversity. There were significant differences in root exudates among different varieties of the same crop (Pang et al., 2021), which may be one of the reasons for the high microbial diversity of drought-tolerant spring wheat in this study.

### 4.2. Drought-induced enrichment of drought-resistant microbial community in spring wheat

Changes in microbial communities between compartments associated with plant roots are mostly governed by the host selectivity, which is influenced by the environment. Therefore, it has a strong driving effect on shaping the root-related microbial community (Chaparro et al., 2014; Reinhold-Hurek et al., 2015; Wagner et al., 2016). This experiment demonstrated that drought stress alters the microbial community composition in the root space of spring wheat. Among them, *Actinobacteriota* and *Variovorax* were significantly enriched in the endosphere, and the relative abundance increased with the deepening of drought stress. The drought-tolerant group strongly preferred *Streptomyces* and other Bacteria, while the drought-sensitive group preferred *Pseudomonas* and other Bacteria. *Variovorax* and *Streptomyces* were significant biomarkers in the roots and rhizosphere of spring wheat under drought stress. Numerous studies have shown that *Actinobacteriota* is widely enriched in numerous plants under drought stress (Naylor and Coleman-Derr, 2017; Santos-Medellín et al., 2017; Fitzpatrick et al., 2018), and *Actinobacteriota* was rapidly enriched in the roots, with the highest relative abundance.

Furthermore, it has been reported that the relative abundance of *Streptomyces* in *Actinobacteriota* continues to rise under drought stress, which may provide long-term memory stress protection for plants after drought alleviation (Santos-Medellín et al., 2021). Our study found that the relative abundance of *Streptomyces* in the endosphere of spring wheat in the drought-tolerant group gradually increased with the deepening of drought, and was significantly higher than that in the drought-sensitive group. This also suggests that *Streptomyces* has a predilection for drought-tolerant types and is more likely to favor them. This conjecture has also been confirmed in the studies of Fitzpatrick et al. (2018) and Santos-Medellín et al. (2021). *Variovorax* can protect plants from drought stress (Qi et al., 2022). Finkel et al. (2020) found that the widely distributed genus *Variovorax* can produce and degrade auxin, which can maintain normal root development through the rhizosphere environment.

Moreover, they further determined the core position of *Variovorax* in the plant-microorganism-microorganism interaction network. *Variovorax* biomarker levels in drought-tolerant and drought-sensitive spring wheat rhizosphere were considerably different. Simultaneously the relative abundance of *Variovorax* was significantly increased under drought stress in the spring wheat’s roots in both groups. Based on our findings and earlier research on the genus *Variovorax*, we believe the genus *Variovorax* may play a significant role in enhancing spring wheat drought tolerance.

We also found that the relative abundance of *Basidiomycota*, *Schizothecium*, and *Conocybe* in spring wheat rhizosphere and bulk soil increased significantly under drought stress. *Parastagonospora* was only enriched in the endosphere, and its abundance increased with the drought stress aggravation. The increased relative abundance of *Conocybe* is beneficial to maize growth under drought stress (Yang et al., 2022). In the experimental studies of long-term rotation (Neupane et al., 2021) and organic fertilizer (Ding et al., 2017), it was found that the relative abundance of *Schizothecium* in soil increased significantly, and the increase of the abundance of the Bacteria could alleviate the harm of other pathogens to crops. *Parastagonospora* is the pathogen of wheat glume blight. It produces cell necrosis in wheat plants carrying the dominant susceptible gene by secreting some effectors, thus providing nutrition for its growth and reproduction (Dagvadorj et al., 2022). Therefore, the effect of drought stress on the fungal community in the spring wheat’s root space will result in the coexistence of beneficial and harmful Bacteria. However, harmful Bacteria appear more inclined to enrich the root.

Additionally, we found that the effect of drought stress on the crop endosphere was greater than on the rhizosphere and bulk soil. We do not have concrete proof that this outcome is related to the enrichment of related diseases such as *Parastagonospora*, which is a problem that must be addressed in future studies on the crop-microorganism collaborative drought resistance mechanism. In summary, the spring wheat’s root space under drought stress will enrich drought-related microbial communities such as *Variovorax*, *Streptomyces*, and *Conocybe*.

### 4.3. Root niche of spring wheat determines the difference in microbial response to drought stress

In this study, the effect of spring wheat root spatial position on bacterial and fungal Alpha diversity was greater than drought treatment. Soil microbial properties were related to the changes of bacterial and fungal communities in the rhizosphere and bulk soil of spring wheat under drought stress. In contrast, endosphere bacterial community composition responded more to soil chemical properties (pH, SOC, TK, and TP). Ladau et al. (2018) showed that soil physical and chemical properties (pH, organic carbon, C: N ratios) were closely related to soil microbial community structure and richness, and the distribution of soil characteristics lagged behind climate change, which in turn led to the lag of Bacteria and Archaea. Different drought treatment methods will affect soil matrix potential and change soil physical properties. In PEG or pot experiments, when the water treatment reached −1.5Mpa, the soil adsorption capacity was large, the plant was at a permanent wilting point, the growth was stagnant, and the microorganisms were difficult to survive. However, for this study, there is a difference in water gradient between soil layers in the field. Whether 0–20 cm of the soil mass water content of 9–12% reaches the permanent wilting point needs to be further judged by plant height, plant physiology, differential genes, and other indicators. Furthermore, (Yang Y. et al., 2021) found that soil microbial biomass was vital in solution under drought stress, which was conducive to the microorganisms’ survival under high osmotic pressure. The species correlation network revealed that the network density of bacterial and fungal communities in the root space of the drought-tolerant spring wheat under drought stress was BS > RS > RE; in the drought-sensitive group, the network density of Bacteria rhizosphere was the highest, and the results of Fungi were consistent with the drought-tolerant group. Xiong et al. (2021) also found that the complexity of the soil network gradually decreased from bulk soil-rhizosphere-endosphere, which was consistent with the results of this study. A rise in drought may result in more antagonistic or competitive biological interactions (Wang et al., 2018; Jiao et al., 2022). The same results were obtained in this study. Drought stress increased the negative correlation between spring wheat’s root space species, which was more intuitive in fungal communities. This may be because drought exacerbates the continual decline of water, which decreases species stability and increases species competition, while species filling the small global network limits the efficiency of resource transfer (Ghoul and Mitri, 2016; Morriën et al., 2017; Jiao et al., 2022). Through the species attributes analysis, it was found that the connectivity number and positive correlation were higher. The root space Bacteria were clustered in *Proteobacteria*, *Actinobacteriota*, *Firmicutes*, *Patescibacteria*, and *Bacteroidota*. The above bacteria may be the key core Bacteria of drought stress.

Functional prediction analysis showed that in Bacteria, the abundance of nitrification and ureolysis in drought-tolerant spring wheat increased significantly in endosphere and bulk soil, respectively. The abundance of nitrogen fixation in group 2 (DTG and DSG) was significantly reduced. In fungi, the symbiotroph forms of DTG and DSG spring wheat (Arbuscular Mycorrhiza) under drought stress increased significantly in the endosphere and bulk soil. Fungal Parasite-Plant Pathogen-Plant Saprotroph both increased significantly in (RE-RS-BS). Nitrification is a crucial part of the soil nitrogen cycle, facilitated by nitrifying Bacteria. In addition to autotrophic nitrifying Bacteria, heterotrophic Bacteria, and *Actinomycetes* can oxidize ammonium salt into nitrite and nitric acid. The heterotrophic bacteria’ ammonium oxidation efficiency is low, but their acid resistance and tolerance to hostile environments are strong. It also contributes to nitrification (Nelson et al., 2016; Kuypers et al., 2018). Microbially induced calcium carbonate precipitation (MICP) can be promoted by ureolysis, which is an innovative method for enzymatic hydrolysis of urea to improve soil (Lauchnor et al., 2015; Hamdan et al., 2017). Xun et al. (2021) showed that functional genes related to soil nitrogen and phosphorus metabolism were the most critical factors in community stability. Bacterial groups represented by nitrogen and phosphorus metabolism may be the core members to maintain the stability of soil microbial communities. Arbuscular mycorrhizal fungi (AMF) are multifunctional symbionts with important ecological significance for most terrestrial plants (Gorzelak et al., 2017; Gu et al., 2020; Jiang et al., 2020; Zhang et al., 2020; Kim et al., 2022). AMF can use fungal hyphae to obtain soil water and nutrients such as phosphorus (P), nitrogen (N), and trace elements for plant absorption (Bulgarelli et al., 2013; Pellegrino et al., 2019; Jiang et al., 2020). The prediction results of microbial community function also showed that Bacteria and Fungi in the spring wheat’s root space under drought stress would respond to drought through different strategies. However, because this study only explored the function of microorganisms at the level of functional prediction, there were still certain limitations, and additional research on microbial functions was still necessary. In conclusion, the changes in the microbial community in the spring wheat’s root space were mainly determined by the niche differentiation of the endosphere, rhizosphere, and bulk soil. Enriching drought-resistant preference groups like *Actinobacteriota*, *Variovorax*, and *Streptomyces* could significantly change the structure and function of the microbial community in spring wheat’s root space under stress.

## 5. Conclusion

This study detected significant differences in bacterial and fungal diversity in the root space of spring wheat under drought stress (*P* < *0.05*). Moreover, the effects on microbial diversity in the endosphere, rhizosphere, and bulk soil gradually weakened. Compared with the drought-sensitive group, the drought-tolerant group had higher microbial diversity. The endosphere microbial community composition was more responsive to drought stress. *Actinobacteriota* and *Streptomyces* preferred the drought-tolerant group, while *Proteobacteria* and *Pseudomonas* preferred the drought-sensitive group. The genus *Variovorax* is vital in assisting spring wheat drought resistance in the rhizosphere and bulk soil. The antagonism between the endosphere and spring wheat species grew, but the community’s complexity and stability diminished. The functional prediction showed that drought stress induced Bacteria to tend to nitrogen cycle (nitrification and ureolysis) related functional taxa enrichment, and Fungi tended to symbiotroph (Arbuscular Mycorrhiza) related functional taxa enrichment. Therefore, under drought stress, Bacteria and Fungi in the spring wheat’s root space may respond with distinct strategies. Of course, these strategies may include the response of microbial network structure to drought, the regulation of microorganisms by host plants, and the resistance of aboveground and underground communities to drought. Future research should further explore the mechanism of crop-soil-microorganism collaborative drought resistance in farmland.

## Data availability statement

The datasets presented in this study can be found in online repositories. The names of the repository/repositories and accession number(s) can be found below: https://www.ncbi.nlm.nih.gov/, PRJNA943479.

## Author contributions

ZL, XiaoZ, YR, and JF contributed to the conception and design of the study. JF and SW carried out field experiments. YC, XianZ, and JW conducted experimental guidance and help. SW, JM, and YG carried out experimental sample collection and detection. GS, LB, and RX performed the statistical analysis. JF wrote the first draft of the manuscript. All authors contributed to manuscript revision and read and approved the submitted version.

## Footnotes

1
https://cloud.majorbio.com

2
http://www.mothur.org/wiki/Calculators

3
http://huttenhower.sph.harvard.edu/LEfSe
