# Supplementary material for: Character variation of root space microbial community composition in the response of drought-tolerant spring wheat to drought stress
Source: Front Microbiol. 2023 Sep 15;14:1235708. doi: 10.3389/fmicb.2023.1235708 (PMC10541208; doi:10.3389/fmicb.2023.1235708)
Supplement: Supplementary file 1 [file Data_Sheet_1.docx]

Supplementary Material

Character variation of root space microbial community composition in the response of drought-tolerant spring wheat to drought stress

Jing Fang^1,3,4^, Shuli Wei^1,2,3,4^, Yanrong Gao^1,3,4^, Xiangqian Zhang^2,3,4^, Yuchen Cheng^2,3,4^, Jianguo Wang^2,3,4^, Jie Ma^1,3,4^, Gongfu Shi^1^, Lanfang Bai^5^, Rui Xie^2,3,4^, Xiaoqing Zhao^1,2.3,4*^, Yongfeng Ren^1,2.3,4*^, Zhanyuan Lu^1,2,3,4*^

*** Correspondence:**

Xiaoqing Zhao

zhaoxq204@163.com

Yongfeng Ren

renyongfeng_1984@163.com

Zhanyuan Lu

lzhy2811@163.com

Table S1 Name and origin of the tested 6 varieties.

| Type | wheat cultivars | Authorized number | Origin |
| --- | --- | --- | --- |
| Drought-tolerant group  (DTG) | Longmai No. 36（LM36） | Heilongjiang  authorized wheat variety  2013001 | Heilongjiang |
|  | Longmai No. 33（LM33） | National authorized wheat variety 2010022 | Heilongjiang |
|  | Dingxi No. 40（DX40） | National authorized wheat variety 2009032 | Gansu |
| Drought-sensitive group  (DSG) | Nongmai No. 2（NM2） | National authorized wheat variety 2006030 | Inner Mongolia |
|  | Bamai No. 12（BM12） | Inner Mongolia authorized wheat variety 2015002 | Inner Mongolia |
|  | Bafeng No. 5（BF5） | National authorized wheat variety 2009028 | Inner Mongolia |

Table S2 Number of high-quality sequences and Mean length for each sample.

| Sample ID | Bacteria | | Fungi | |
| --- | --- | --- | --- | --- |
|  | Number of high-quality sequences | Average Length | Number of high-quality sequences | Average Length |
| RE_CKDTG | 1136739 | 252.90 | 1490654 | 236.05 |
| RE_CKDSG | 1511069 | 252.76 | 1345242 | 252.57 |
| RE_MDDTG | 1172027 | 252.94 | 1496220 | 244.04 |
| RE_MDDSG | 1315849 | 252.87 | 1429249 | 242.03 |
| RE_SDDTG | 1323782 | 252.99 | 1032060 | 246.15 |
| RE_SDDSG | 1074320 | 252.90 | 1113039 | 244.73 |
| RS_CKDTG | 1354446 | 253.12 | 1143055 | 241.01 |
| RS_CKDSG | 1445177 | 253.13 | 1152408 | 245.21 |
| RS_MDDTG | 1472447 | 253.08 | 1382862 | 241.14 |
| RS_MDDSG | 1228763 | 253.10 | 1259980 | 244.69 |
| RS_SDDTG | 1123288 | 253.08 | 1318433 | 242.95 |
| RS_SDDSG | 1352802 | 253.08 | 1417045 | 252.19 |
| BS_CKDTG | 1348077 | 253.14 | 1044293 | 242.58 |
| BS_CKDSG | 1240540 | 253.14 | 1030255 | 245.03 |
| BS_MDDTG | 1440575 | 253.09 | 1184998 | 249.43 |
| BS_MDDSG | 1271110 | 253.11 | 1141680 | 251.40 |
| BS_SDDTG | 1098838 | 253.08 | 1154227 | 247.95 |
| BS_SDDSG | 1649342 | 253.09 | 1021857 | 253.38 |
| Total | 23559191 |  | 22157557 |  |

Table S3 Effects of drought stress on bacterial and fungal alpha diversity (Shannon index).

| Type |  | Bacterial  Shannon index | Fungal  Shannon index |
| --- | --- | --- | --- |
| Endosphere | P  F | 0.48  0.74 | 0.30  1.23 |
| Rhizosphere | P  F | <0.01  30.17 | 0.68  0.38 |
| Bulk soil | P  F | <0.01  8.06 | 0.11  2.36 |
| Drought treatment | P  F | <0.01  5.58 | 0.42  0.88 |
| Variety category | P  F | 0.34  0.94 | 0.83  0.05 |
| Spatial position | P  F | <0.01  1024.98 | <0.01  325.81 |
| Drought treatment×Variety category | P  F | 0.21  1.58 | 0.19  1.69 |
| Drought treatment×Spatial position | P  F | 0.91  0.25 | 0.42  0.99 |
| Variety category×Spatial position | P  F | 0.02  4.26 | 0.65  0.43 |
| Drought treatment×Variety category×Spatial position | P  F | 0.94  0.19 | <0.01  3.63 |

Note: Data analysis was performed by One-way ANOVA and linear mixed model(LMM).

Table S4 PERMANOVA test for the difference of microbial community structure in root space of spring wheat under different drought treatments.

| Type |  | Bacterial community | Fungal community |
| --- | --- | --- | --- |
| Endosphere | P  R^2^ | <0.001  0.34 | <0.001  0.28 |
| Rhizosphere | P  R^2^ | <0.001  0.39 | <0.001  0.45 |
| Bulk soil | P  R^2^ | <0.001  0.35 | <0.001  0.54 |
| Drought treatment | P  R^2^ | <0.001  0.05 | <0.001  0.17 |
| Variety category | P  R^2^ | 0.37  0.01 | <0.001  0.02 |
| Spatial position | P  R^2^ | <0.001  0.64 | <0.001  0.26 |
| Drought treatment×Variety category | P  R^2^ | <0.001  0.07 | <0.001  0.19 |
| Drought treatment×Spatial position | P  R^2^ | <0.001  0.72 | <0.001  0.45 |
| Variety category×Spatial position | P  R^2^ | <0.001  0.65 | <0.001  0.28 |
| Drought treatment×Variety category×Spatial position | P  R^2^ | <0.001  0.77 | <0.001  0.55 |

Table S5 Variance partitioning analysis (VPA) of the effects of soil chemical and microbial properties on bacterial and fungal communities in root space (RE, RS, BS) of spring wheat.

| From | To | endosphere | rhizosphere | bulk soil |
| --- | --- | --- | --- | --- |
| Bacteria |  |  |  |  |
| SMP | Bacteria | 2.90% | 13.24% | 14.60% |
| SCP | Bacteria | 3.54% | 7.46% | 2.99% |
| SMP+SCP | Bacteria | 3.53% | 4.19% | 6.28% |
| Residuals | | 90.04% | 75.12% | 76.13% |
| Fungi |  |  |  |  |
| SMP | Fungi | 4.69% | 16.35% | 9.97% |
| SCP | Fungi | 3.79% | 7.00% | 7.84% |
| SMP+SCP | Fungi | 1.76% | 1.97% | 12.50% |
| Residuals | | 89.76% | 74.68% | 69.69% |

Note: soil chemical properties (SCP); soil microbial properties (SMP).

Table S6 Soil environmental factors of spring wheat field under drought stress.

| soil environment factor | CK_DTG | MD_DTG | SD_DTG | CK_DSG | MD_DSG | SD_DSG |
| --- | --- | --- | --- | --- | --- | --- |
| pH | 7.07±0.02d | 7.29±0.02b | 7.37±0.02a | 7.18±0.03c | 7.11±0.01d | 7.28±0.02b |
| SOC  (g·kg^−1^) | 33.97±0.64a | 30.72±0.59b | 25.91±0.80d | 34.11±1.00a | 28.35±0.42c | 23.33±0.65e |
| TP  (g·kg^−1^) | 3.94±0.07bc | 4.05±0.04ab | 4.12±0.05a | 3.76±0.03d | 3.91±0.02c | 4.04±0.03ab |
| TK  (g·kg^−1^) | 13.34±0.18d | 15.70±0.26c | 18.22±0.34b | 13.51±0.22d | 17.82±0.22b | 19.12±0.22a |
| CAT  (mg·g^−1^) | 39.84±0.21d | 40.33±0.16c | 40.75±0.08b | 38.68±0.13e | 40.87±0.20b | 41.31±0.07a |
| SC  (mg·g^−1^) | 27.51±0.02a | 27.37±0.03b | 27.01±0.05c | 27.45±0.03ab | 27.10±0.08c | 26.80±0.04d |
| URE  (mg·g^−1^) | 4.64±0.08b | 5.02±0.03a | 5.10±0.06a | 4.75±0.08b | 5.12±0.09a | 5.16±0.04a |
| ALP  (mg·g^−1^) | 0.287±0.003a | 0.281±0.002ab | 0.272±0.003cd | 0.288±0.003a | 0.278±0.002bc | 0.265±0.001d |
| MBC  (mg·kg^−1^) | 113.32±3.84b | 83.04±5.43c | 40.19±4.04d | 125.96±7.15a | 51.55±7.83d | 44.80±1.43d |
| MBN  (mg·kg^−1^) | 123.17±5.80bc | 17.77±3.82c | 87.38±2.88d | 173.99±10.16a | 127.03±9.56b | 106.99±5.76c |
| MBP  (mg·kg^−1^) | 17.27±1.34b | 14.29±1.25c | 11.00±0.67d | 23.29±0.82a | 12.07±1.46cd | 6.18±0.97e |

Note: CK: control treatment; MD: Moderate drought treatment; SD: Severe drought treatment. DTG: Drought-tolerance group, Including three spring wheat varieties (DX40, LM36, and LM33). DSG: Drought-sensitive group, Including three spring wheat varieties (NM2, BM12, and BF5). The index values of soil environmental factors in the spring wheat field were the mean values of 9 values with 3 replicates in 3 spring wheat varieties (mean ± standard error). Soil chemical properties include pH; SOC, soil organic carbon content; TP, total phosphorus content; TK, total potassium content; soil microbiological properties include CAT, soil catalase activity; SC, soil invertase activity; URE, soil urease activity; ALP, soil alkaline phosphatase activity; MBC, soil microbial biomass carbon; MBN, soil microbial biomass nitrogen; MBP, soil microbial biomass phosphorus. The lowercase letters indicated that the mean values of soil environmental factors were significantly different (P < 0.05, ANOVA).

Table S7 Topological indices of each network in Fig. 8.

| Classification levels | Group | Clustering coefficient | Transitivity | Network density | Number of nodes | Netework heterogeneity | Netework centralization |
| --- | --- | --- | --- | --- | --- | --- | --- |
| Bacteria | RE_DTG | 0.504 | 0.540 | 0.165 | 29 | 0.618 | 0.206 |
|  | RE_DSG | 0.514 | 0.506 | 0.202 | 30 | 0.633 | 0.264 |
|  | RS_DTG | 0.582 | 0.561 | 0.320 | 29 | 0.461 | 0.347 |
|  | RS_DSG | 0.737 | 0.782 | 0.483 | 29 | 0.464 | 0.325 |
|  | BS_DTG | 0.758 | 0.707 | 0.485 | 29 | 0.390 | 0.313 |
|  | BS_DSG | 0.569 | 0.625 | 0.308 | 29 | 0.493 | 0.245 |
| Fungi | RE_DTG | 0.330 | 0.441 | 0.127 | 28 | 0.757 | 0.262 |
|  | RE_DSG | 0.261 | 0.319 | 0.147 | 20 | 0.746 | 0.363 |
|  | RS_DTG | 0.537 | 0.504 | 0.220 | 28 | 0.531 | 0.362 |
|  | RS_DSG | 0.501 | 0.610 | 0.219 | 29 | 0.684 | 0.340 |
|  | BS_DTG | 0.699 | 0.720 | 0.428 | 26 | 0.553 | 0.317 |
|  | BS_DSG | 0.627 | 0.785 | 0.440 | 26 | 0.538 | 0.347 |

Table S8 Monitoring of soil mass water content in 0-20cm soil layer under drought stress.

| Date | CK (0-20cm) | MD （0-20cm） | SD （0-20cm） |
| --- | --- | --- | --- |
| 4-Jul-2022 | 21.2% | 21.5% | 20.3% |
| 8-Jul-2022 | 31.3% | 18.4% | 17.9% |
| 12-Jul-2022 | 28.8% | 15.3% | 14.9% |
| 16-Jul-2022 | 25.0% | 20.6% | 13.3% |
| 20-Jul-2022 | 30.0% | 19.0% | 12.2% |
| 24-Jul-2022 | 28.4% | 17.5% | 11.5% |
| 28-Jul-2022 | 27.5% | 16.8% | 10.7% |
| 1-Aug-2022 | 26.6% | 16.1% | 10.1% |
| 2-Aug-2022 | 25.9% | 15.4% | 9.6% |

Note: Soil mass water content changes under different treatments. CK treatment was replenished twice (on July 4 and July 16), and the amount of water replenished was 1 m^3^ for each plot. MD treatment was replenished once (July 12), and the amount of water replenished was 0.5 m^3^ for each plot.


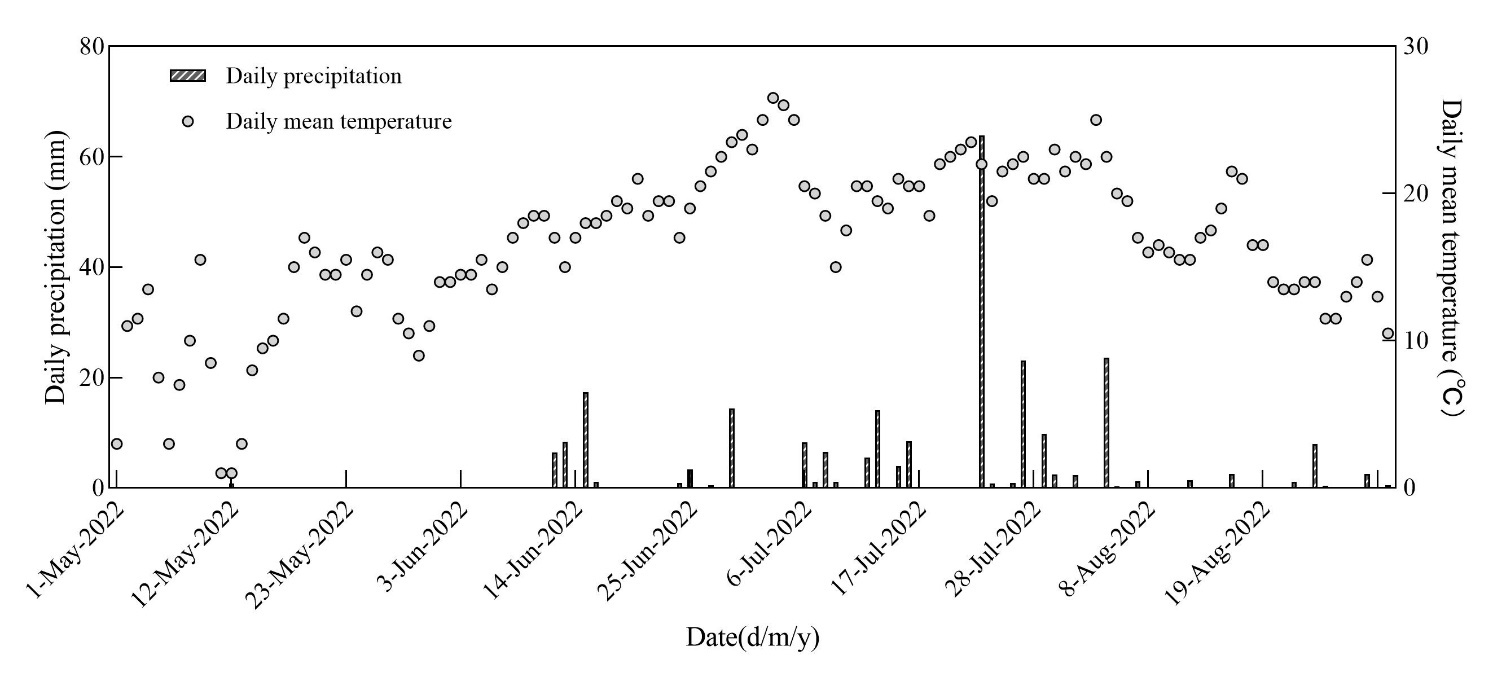


Fig. S1 Distribution map of daily precipitation and daily mean temperature during the whole growth period of spring wheat in 2022


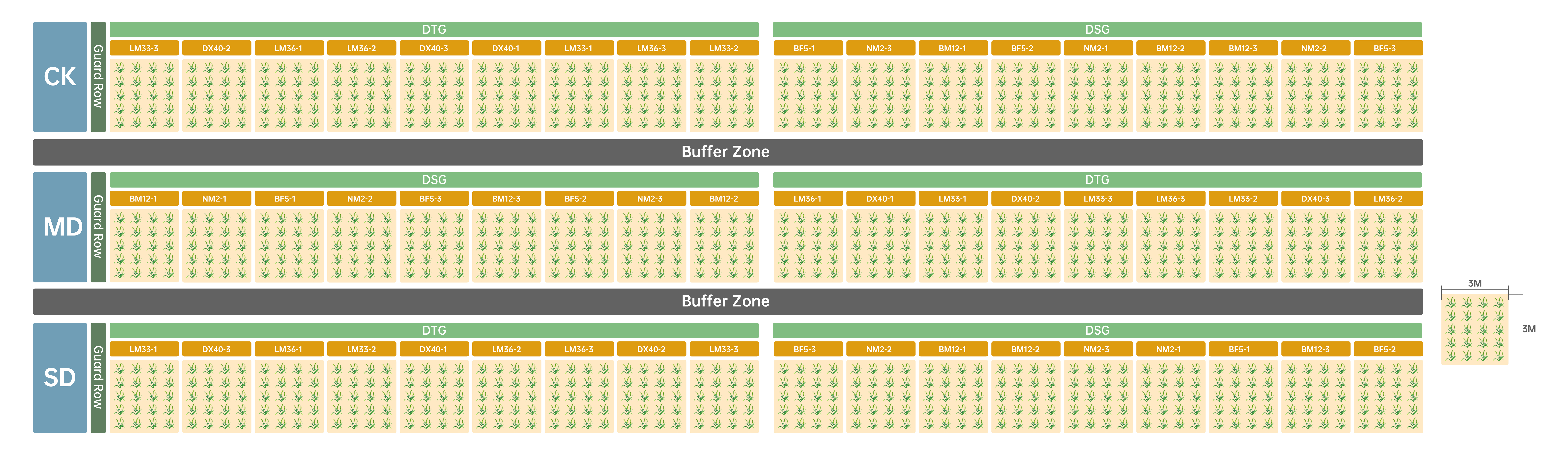


Fig. S2 The layout of the 2022 spring wheat sowing community


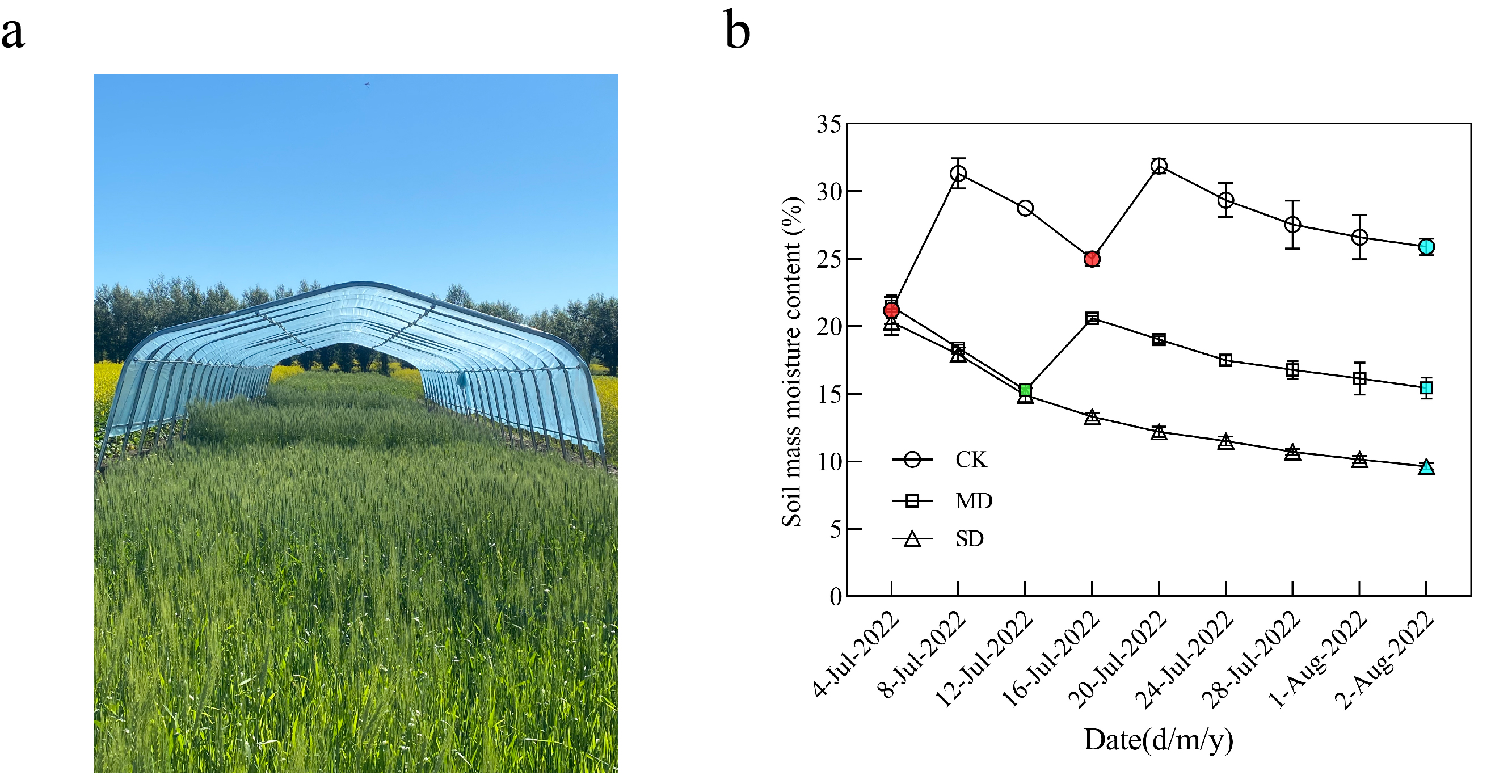


Fig. S3 Daily precipitation distribution map of the whole growth period of spring wheat in 2022

Note: a: Drought shed; b: Soil mass water content changes under different treatments. The red circle in the figure indicates that the CK treatment was replenished twice (on July 4 and July 16), and the amount of water replenished was 1 m^3^ for each plot. The green square in the figure indicates that the MD treatment was replenished once (July 12), and the amount of water replenished was 0.5 m^3^ for each plot. The blue mark is the soil mass water content when sampling on August 2.


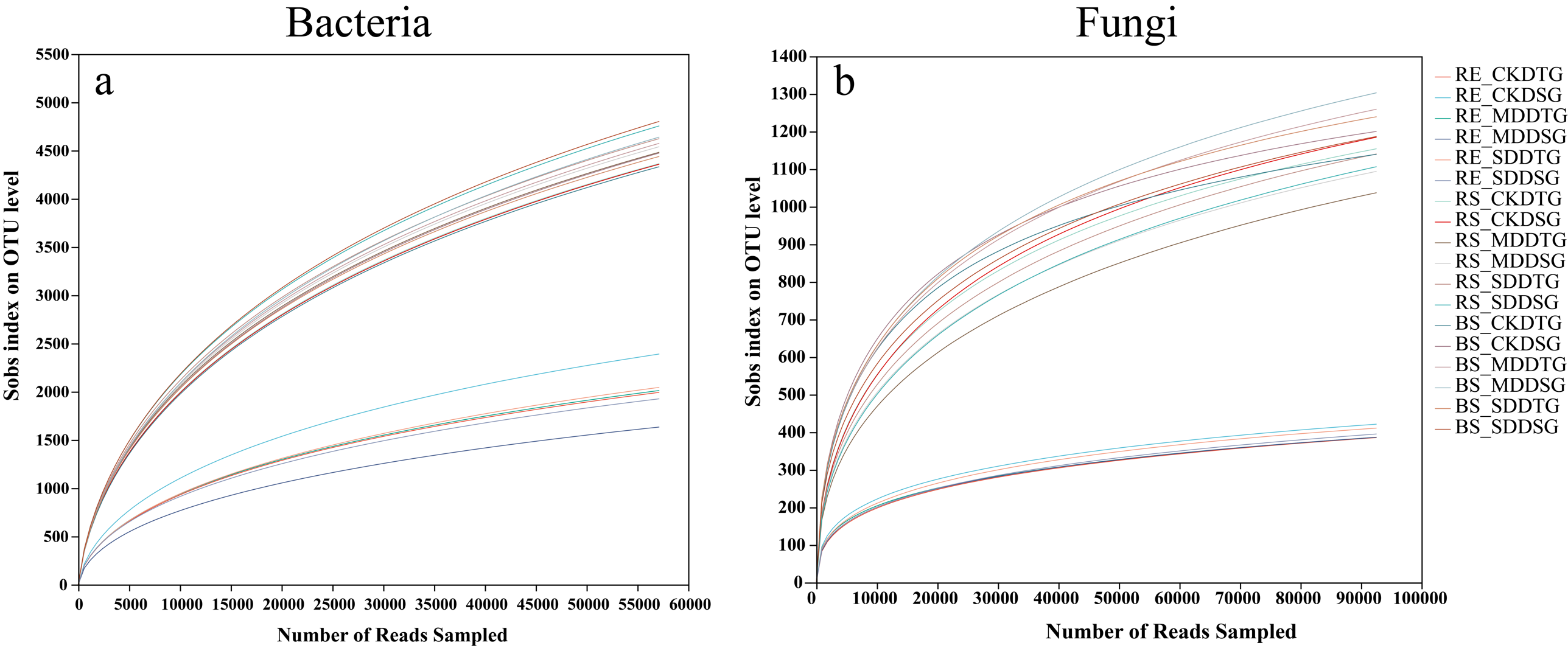
Fig. S4 OTU rarefaction curves of bacteria and fungi in spring wheat root space under different drought treatments

Note: a: Bacteria OTU rarefaction curves at 3 positions (RE, RS and BS); b: Fungi OTU rarefaction curves at 3 positions (RE, RS, BS).


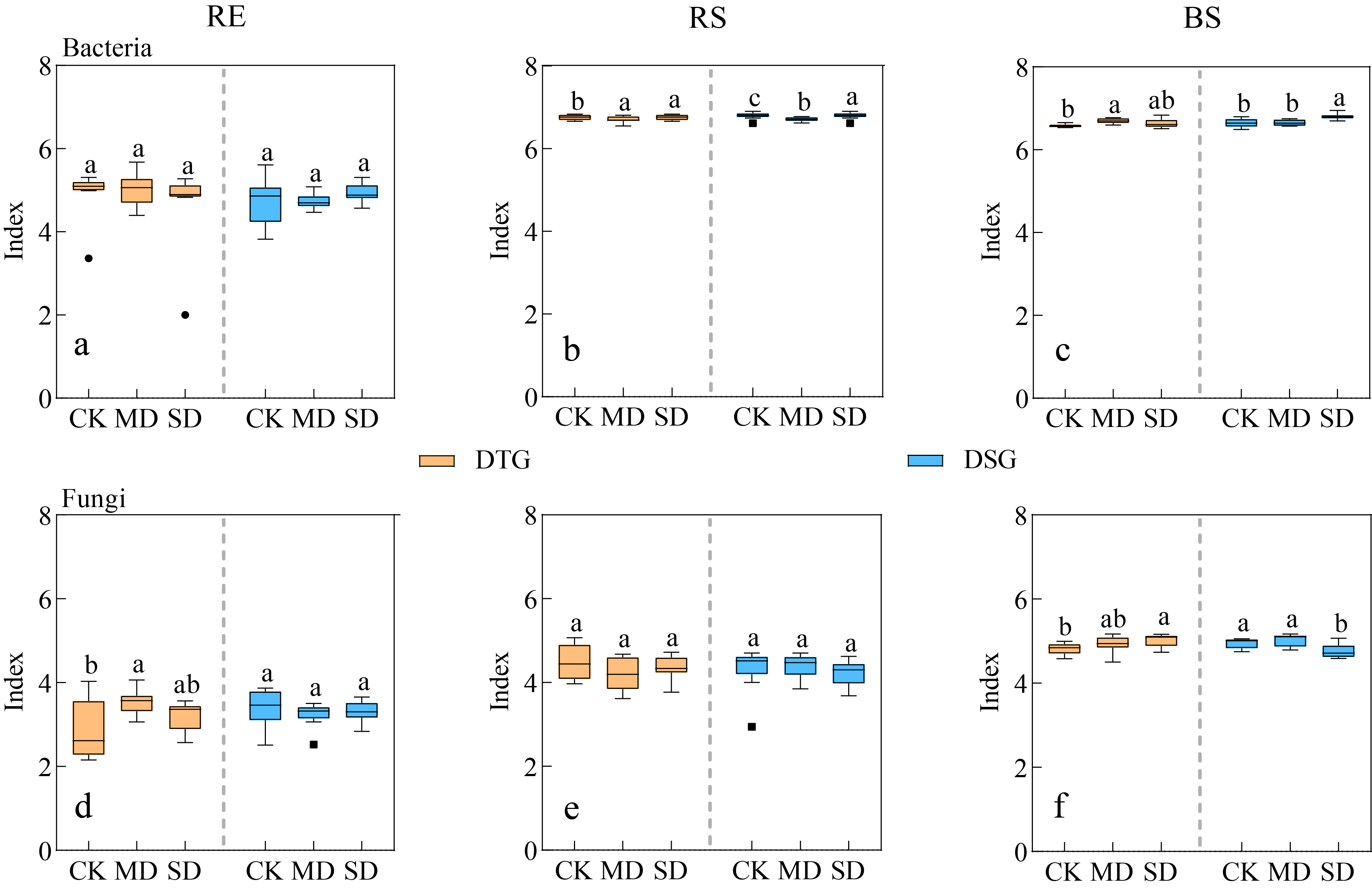


Fig. S5 Shannon diversity index of bacteria and fungi at root space (RE, RS, BS) of spring wheat under different drought treatments

Note: a: Shannon index of RE bacteria; b: Shannon index of RS bacteria; c: Shannon index of BS bacteria; d: Shannon index of RE fungi; e: Shannon index of RS fungi; f: Shannon index of BS fungi.


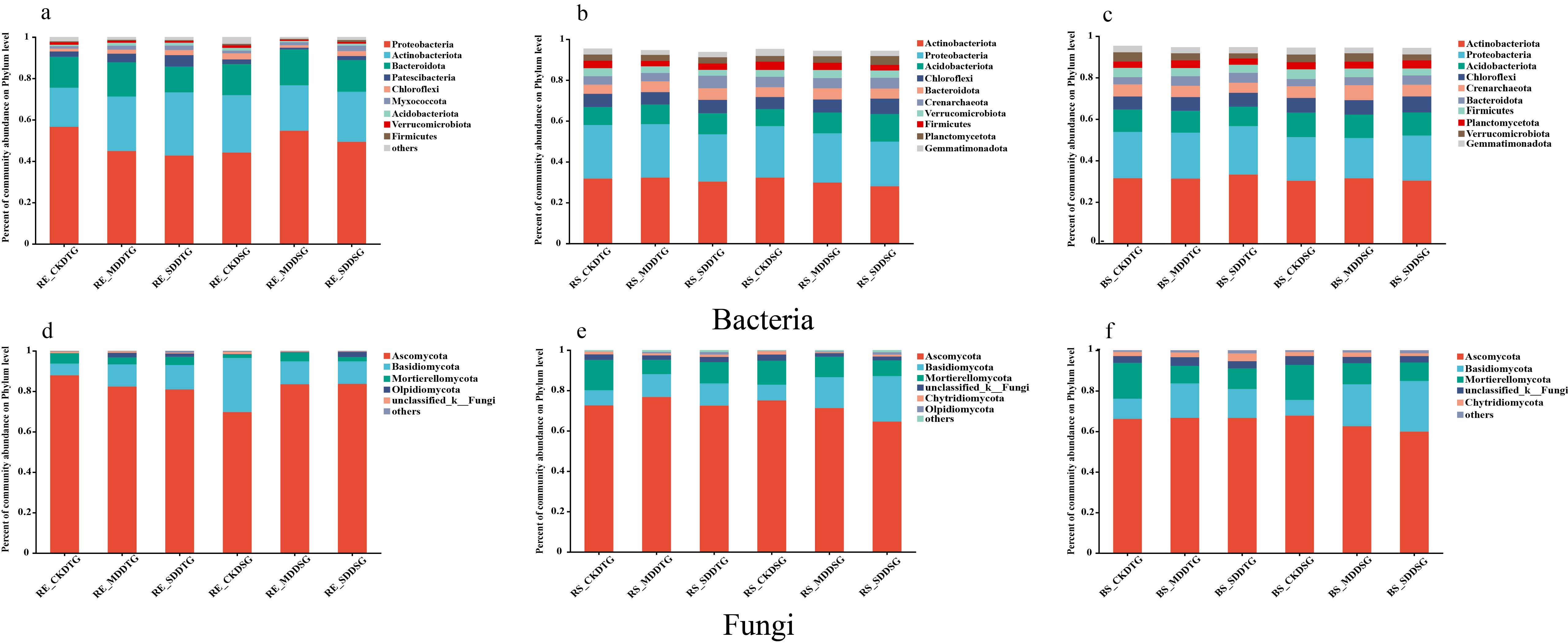


Fig. S6 The relative abundance of bacterial and fungal phylum in root space (RE, RS, BS) of spring wheat under different drought treatments

Note: a: Relative abundance of endosphere (RE) bacterial phylum; b: Relative abundance of the rhizosphere (RS) bacterial phylum; c: Relative abundance of bulk soil (BS) bacterial phylum; d: Relative abundance of endosphere (RE) fungal phylum; e: Relative abundance of the rhizosphere (RS) fungal phylum; f: Relative abundance of bulk soil (BS) fungal phylum.


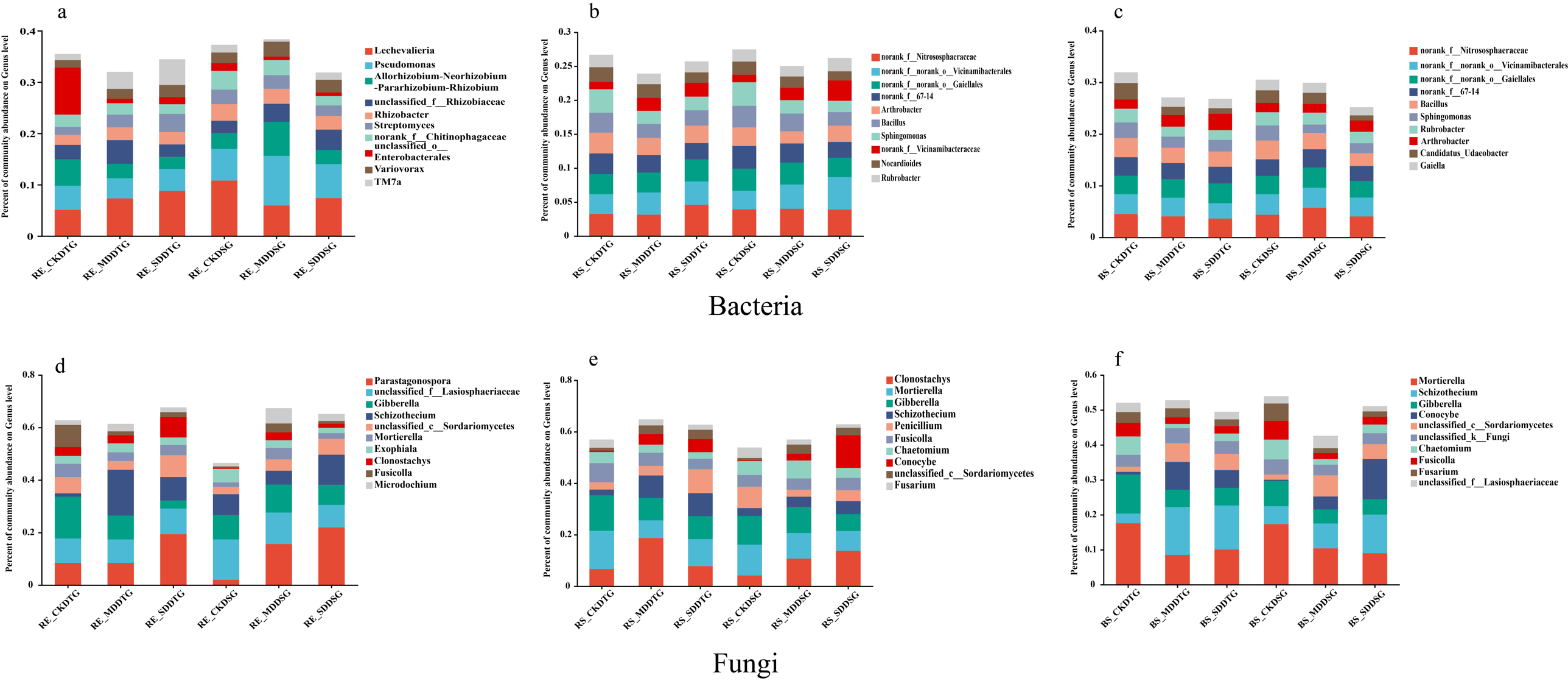


Fig. S7 The relative abundance of bacterial and fungal genus in root space (RE, RS, BS) of spring wheat under different drought treatments

Note: a: Relative abundance of endosphere (RE) fungal genus; b: Relative abundance of the rhizosphere (RS) fungal genus; c: Relative abundance of the bulk soil (BS) fungal genus; d: Relative abundance of the endosphere (RE) fungal genus; e: Relative abundance of the rhizosphere (RS) fungal genus; f: Relative abundance of the bulk soil (BS) fungal genus.


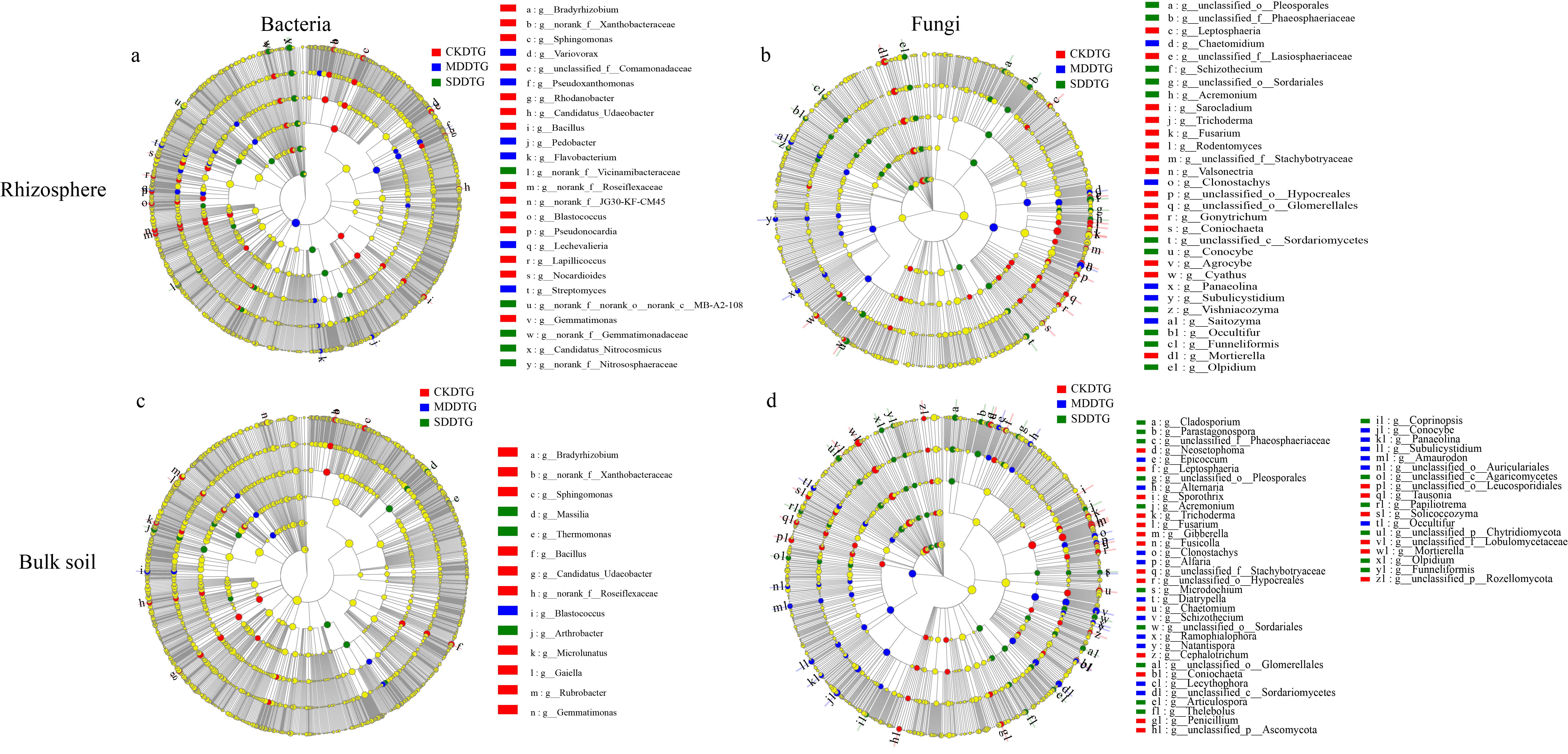


Fig. S8 LEfSe analysis of different species between bacteria (a,c) and fungi (b,d) in the rhizosphere and bulk soil of the drought-tolerant group (DRG) spring wheat under drought treatment

Note: Following the species at the phylum, class, order, family, and genus levels is a cladogram from the inner circle to the outer circle. The figure's letters indicate the species names in the right legend. Different color nodes represent the microbial populations significantly enriched by different treatments, and the differences between groups are significant. The yellow nodes represented the microbial populations with no significant difference between groups (*P < 0.05*, LDA score 3.2).


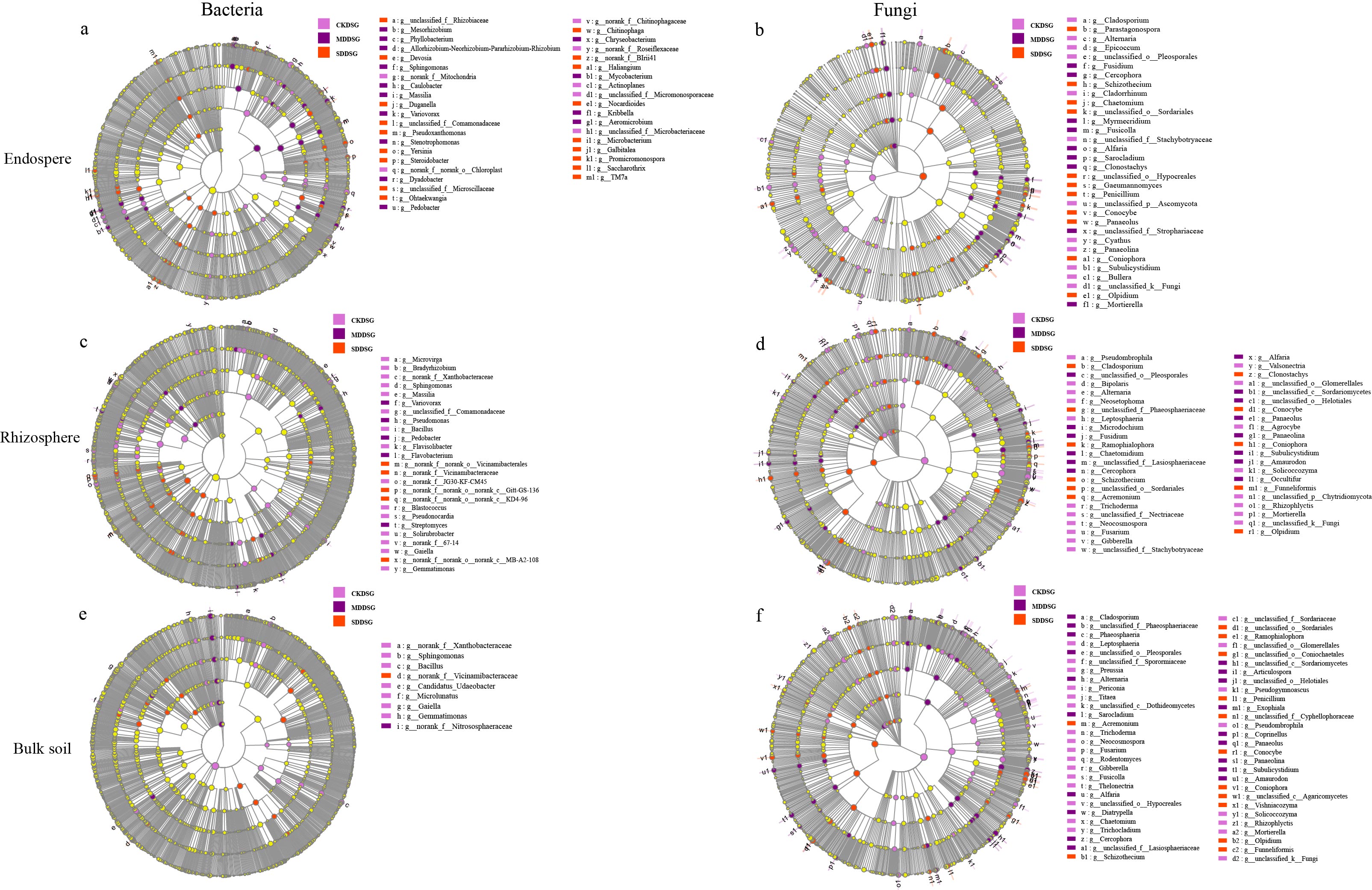


Fig. S9 LEfSe analysis of different species between bacteria (ace) and fungi (bdf) in root space of the drought-sensitive group (DSG) spring wheat under drought treatment

Note: Following the species at the phylum, class, order, family, and genus levels is a cladogram from the inner circle to the outer circle. The figure's letters indicate the species names in the right legend. Different color nodes represent the microbial populations significantly enriched by different treatments, and the differences between groups are significant. The yellow nodes represented the microbial populations with no significant difference between groups (*P < 0.05*, LDA score 3.2).


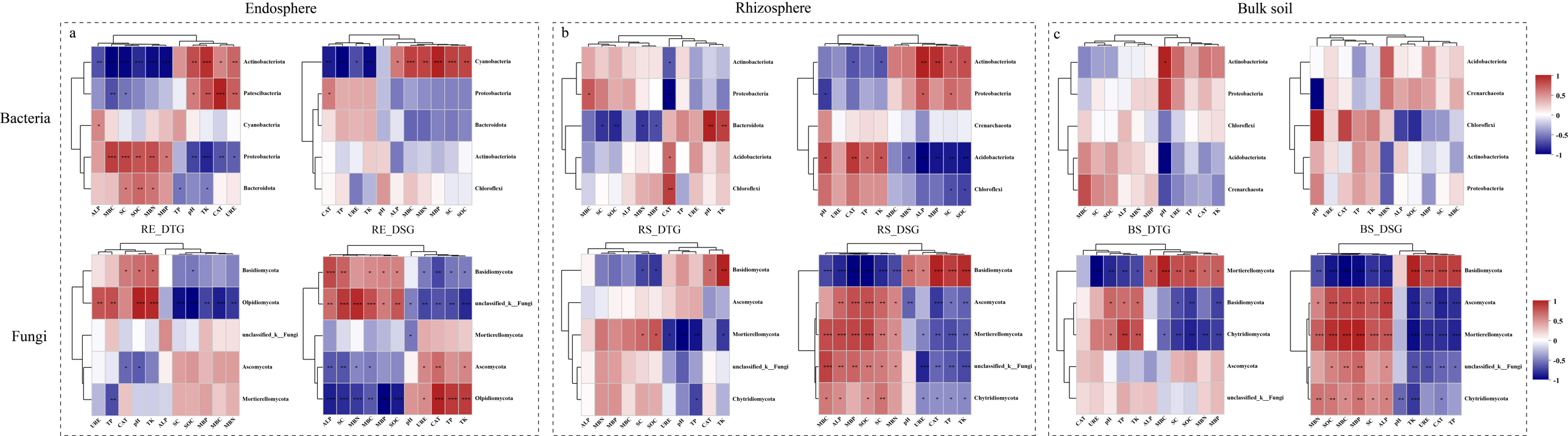


Fig. S10 The drought stress (CK, MD, SD), the correlation heat map of the relative abundance of the dominant enriched phylum of root space microorganisms (RE, RS, BS) in a drought-tolerant Group (DTG) and drought-sensitive Group (DSG) of spring wheat with soil chemical properties and soil microbiological properties

Note: R-values are shown in different colors in the figure. (* *P < 0.05*, ** *P < 0.01*, *** P *≤ 0.001*).


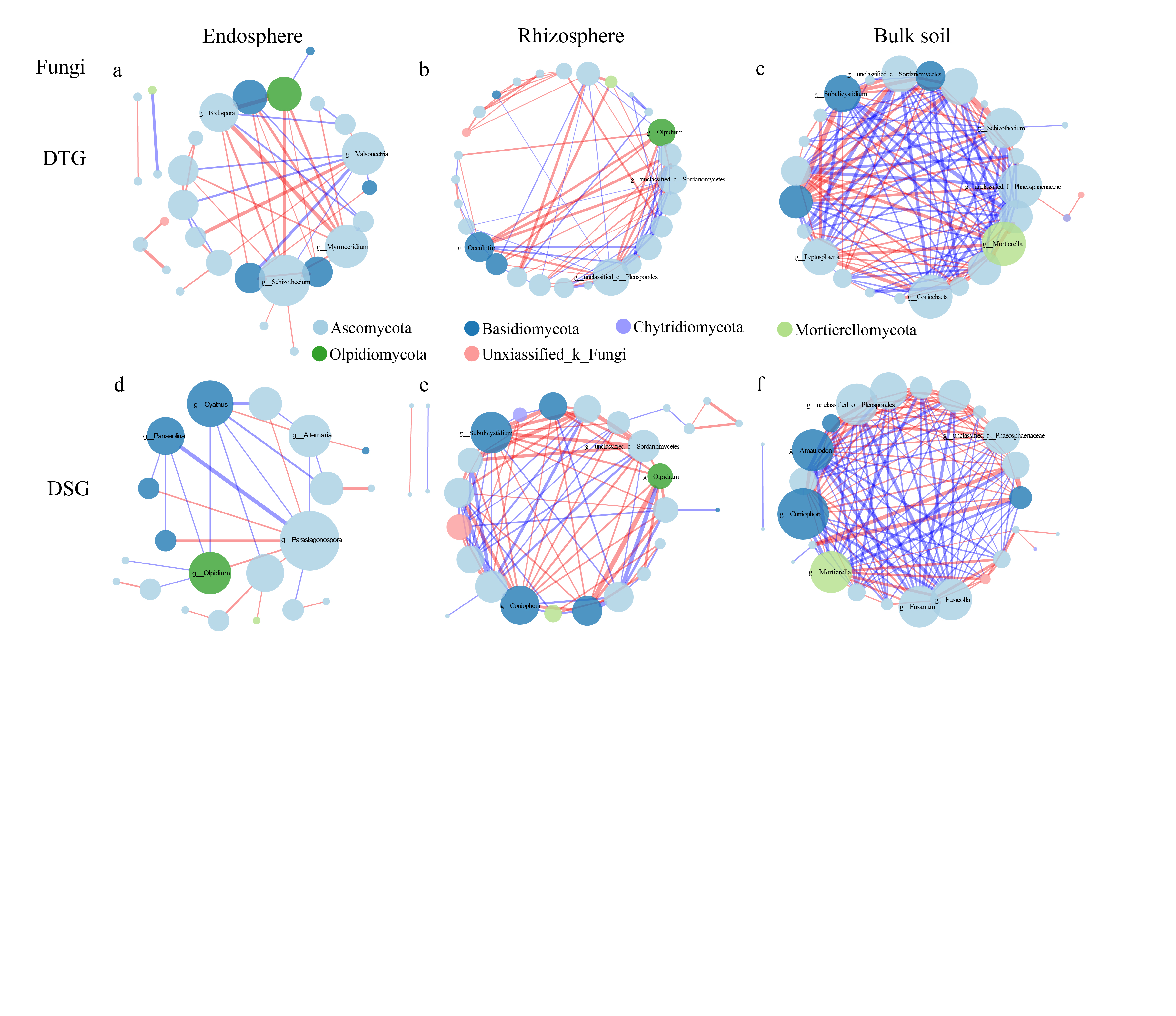
Fig. S11 The correlation network diagram of fungal genus level in two groups (DTG, DSG) of spring wheat under drought stress

Note: The red connection indicates a positive correlation, the blue connection indicates a negative correlation, the connection thickness represents the correlation coefficient size, and the number of lines indicates the degree of connection between nodes.


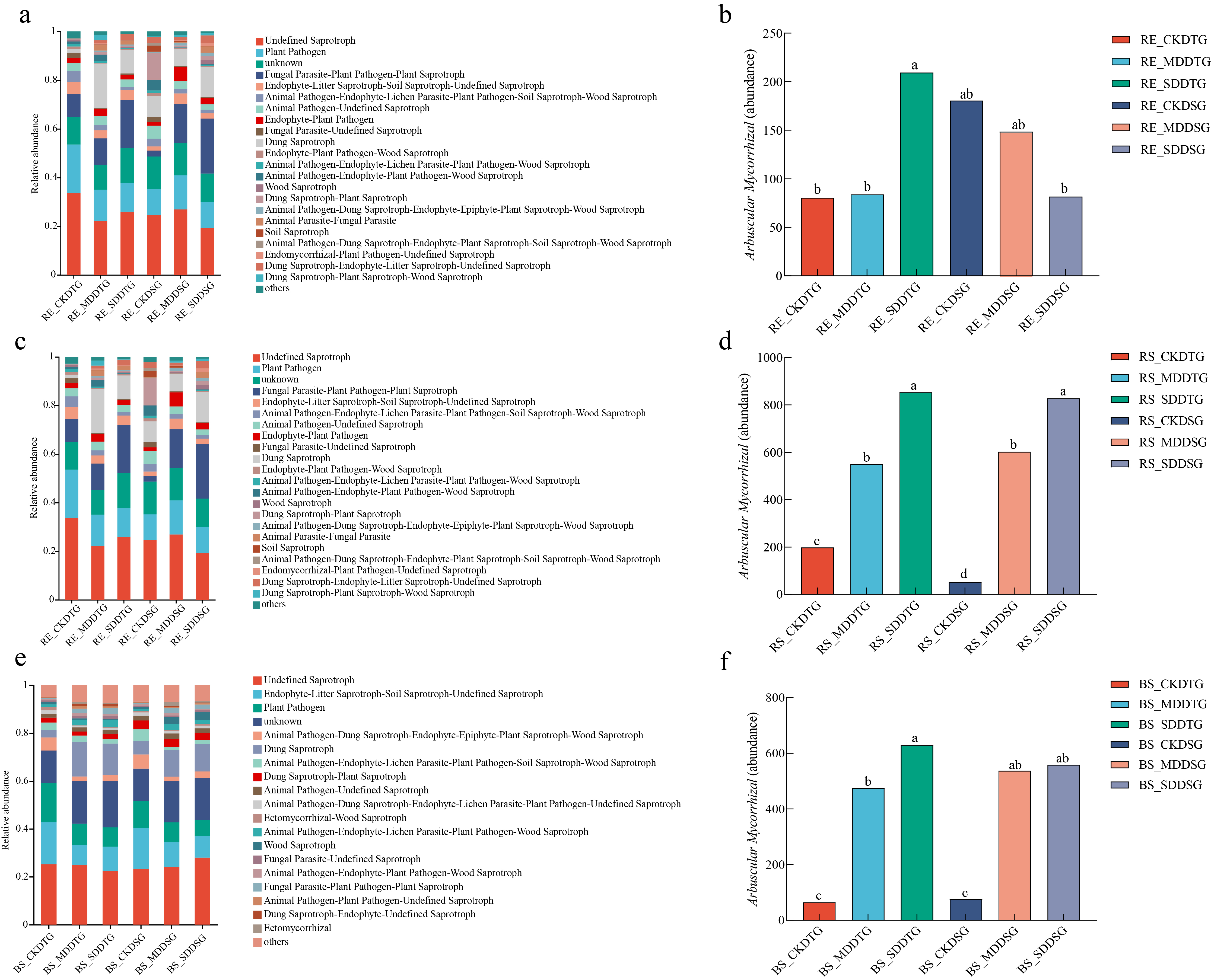


Fig. S12 FUNGuild was used to predict the function of fungal community in root space of spring wheat under drought stress

Note: a, b: Functional prediction of endosphere fungi; c, d: Functional prediction of rhizosphere fungi; e, f: Functional prediction of bulk soil fungi.
